# CDC2 Mediates Progestin Initiated Endometrial Stromal Cell Proliferation: A PR Signaling to Gene Expression Independently of Its Binding to Chromatin

**DOI:** 10.1371/journal.pone.0097311

**Published:** 2014-05-23

**Authors:** Griselda Vallejo, Alejandro D. La Greca, Inti C. Tarifa-Reischle, Ana C. Mestre-Citrinovitz, Cecilia Ballaré, Miguel Beato, Patricia Saragüeta

**Affiliations:** 1 Instituto de Biología y Medicina Experimental, IByME-Conicet, Buenos Aires, Argentina; 2 Centre de Regulació Genòmica, (CRG), Barcelona, Spain; 3 University Pompeu Fabra (UPF), Barcelona, Spain; Baylor college of Medicine, United States of America

## Abstract

Although non-genomic steroid receptor pathways have been studied over the past decade, little is known about the direct gene expression changes that take place as a consequence of their activation. Progesterone controls proliferation of rat endometrial stromal cells during the peri-implantation phase of pregnancy. We showed that picomolar concentration of progestin R5020 mimics this control in UIII endometrial stromal cells via ERK1-2 and AKT activation mediated by interaction of Progesterone Receptor (PR) with Estrogen Receptor beta (ERb) and without transcriptional activity of endogenous PR and ER. Here we identify early downstream targets of cytoplasmic PR signaling and their possible role in endometrial stromal cell proliferation. Microarray analysis of global gene expression changes in UIII cells treated for 45 min with progestin identified 97 up- and 341 down-regulated genes. The most over-represented molecular functions were transcription factors and regulatory factors associated with cell proliferation and cell cycle, a large fraction of which were repressors down-regulated by hormone. Further analysis verified that progestins regulate *Ccnd1, JunD, Usf1, Gfi1, Cyr61,* and *Cdkn1b* through PR-mediated activation of ligand-free ER, ERK1-2 or AKT, in the absence of genomic PR binding. ChIP experiments show that progestin promoted the interaction of USF1 with the proximal promoter of the *Cdc2* gene. *Usf1* knockdown abolished *Cdc2* progestin-dependent transcriptional regulation and cell proliferation, which also blocked *Cdc2* knockdown. We conclude that progestin-induced proliferation of endometrial stromal cells is mediated by ERK1-2 and AKT dependent early regulation of USF1, which directly induces *Cdc2*. To our knowledge, this is the first description of early target genes of progestin-activated classical PR via crosstalk with protein kinases and independently of hormone receptor binding to the genomic targets.

## Introduction

Ovarian steroids are considered to act mainly through direct regulation of transcription via interaction of their receptors with target genes [1], but rapid effects of steroids independent of transcriptional responses have been reported in different tissues and cellular types [2]–[4]. Steroid hormones are able to rapidly and transiently activate the SRC/RAS/ERK kinases cascade through a direct interaction between cytoplasmic steroid receptors with SRC [3], [5]–[6]. This activation is essential for some physiologic responses to hormones, such as cell proliferation or inhibition of apoptosis [7]. Moreover, estrogen receptor alpha (ERa) also interacts with the regulatory subunit of the phosphoinositol-3- kinase, leading to the ER activation as well as the activation of AKT [8]. Activation of cytoplasmic cascades could be involved in the transcriptional regulation of some ovarian hormone target genes. RSK2 kinase, which interacts with the hormone binding domain of ERa and phosphorylates it at Ser 167 [9], can also phosphorylate histone H3 at S10 and thus participate in gene activation. A direct connection between rapid kinase activation and gene induction by steroid hormones has been reported in breast cancer cells. The activation of ERK and MSK1 and their recruitment, along with phosphorylated PR (pPR) to the MMTV promoter leads to phosphorylation of histone H3 at S10, displacement of HP1g, and recruitment of ATP-dependent remodeling complexes, coactivators, and RNA polymerase II [10]. These results show that the cytoplasmic and nuclear pathways activated by steroid hormones converge on chromatin to enable gene regulation in T47D cells.

The uterine endometrium undergoes cyclic stages of proliferation, differentiation and remodelling under the control of steroid hormones. Even though proliferation of endometrial stromal cells followed by differentiation into decidual cells is dependent on progesterone and estradiol [11], [12], a dominant role of progesterone during decidualization has been demonstrated using antiprogestins [13], [14]. Progesterone alone is able to induce uterine stromal proliferation before decidualization, an effect potentiated by estrogens [15]. Evidence that progesterone receptor (PR) is crucial for decidualization comes from PR-deficient mice [16]. Our previous studies in the UIII stromal cell line derived from rat endometrium concluded that induction of proliferation upon the addition of progestins requires both progesterone and estrogen receptor beta (ERb) [17]. The interaction of both receptors in the cytoplasm is needed to activate the extracellular signal-regulated kinases 1 and 2 (ERK1-2) as well as the AKT signaling pathway. UIII cells do not express ERalpha but express PR and ERbeta though at levels insufficient for hormonal transactivation of their respective target genes via binding to genomic target sequences [17]. However, whether the progestin action via kinase signaling can regulate genes independently of PR binding to chromatin remains an open question. In this study we used UIII cell line to explore the cohort of early regulated genes by the cytoplasmic component of progesterone-PR pathway independent of PR binding to genomic targets. We describe early downstream targets of progestin-dependent ERK and AKT activation via PR and ER, and study their role in stromal endometrial proliferation in cultured UIII cells.

## Materials and Methods

### Materials

Available at SI M&M.

### Cell Culture and Hormone Treatment Experiments

UIII rat normal uterine stromal cells were kindly provided by Dr. Cohen and maintained in M199 medium supplemented with 10% fetal bovine serum (FBS) and gentamycin (100 µg/ml) at 37°C in humidified 95% air with 5% CO as the authors first described them [18]. Culture media were changed every 2 days.

For hormone treatment experiments in absence of serum, cells were cultured in FBS and, 48 h later, media were replaced by fresh M199 without serum. After three days in serum-free conditions, media were replaced by either vehicle or hormones.

### RNA Extraction, sqPCR and qPCR Analysis

In all cases total RNA isolation and cDNA synthesis were performed as described [17].

SqPCR and qPCR: *JunD*, *Usf1*, *Cyr61*, *Pten*, *Cdkn1b*, *Crebbp*, *Gfi1* and *β-actin* mRNA levels were quantified as described [19]. The primers used are detailed in Table S1. Find details of these protocols in SI M&M.

### Microarray Analysis

Serum starved UIII cells were treated with ethanol or R5020 10^−10^ M during 45 minutes. Isolated RNA was hybridized to an oligo microarray (60 mer) from Agilent (G4130). cDNA was synthesized according to manufacturer’s instructions (Agilent). Detailed protocols are available at www.agilent.com/chem/dnamanuals-protocols. Briefly, the cDNA was used as a template for synthesis, amplification and staining of cRNA. The dCTP conjugated to cy3 or conjugated to cy5 was incorporated by T7 RNA polymerase to obtain cRNA-cy3 or cRNA-cy5 from the cDNA vehicle or progestin treated cells respectively. The first experiment was performed with an inverted dye swap staining (indicated as DS in figure legend). The cRNA-cy3 and cRNA-cy5 were purified before chip hybridization. The images of competitive resulting hybridization were scanned and data from images were extracted to quantify gene expression on each spot. The data analysis was performed with AFM 4.0 [20]. Microarray analysis was performed at the Microarray unit from the Centre de Regulació Genòmica, Barcelona, Spain. The dataset was reported to GEO databank under GSE55992 accession number.

### Statistical Analysis for Microarrays Data

The details of experimental design, transformation and statistical treatment of microarray data protocols are available at SI M&M.

### In Silico Analysis

In silico analysis was performed using GO Tree Machine and OntoExpress softwares. Details of the analysis in SI M&M.

The DNA sequence corresponding to the PR binding site in *Cdc2* promoter from T47D human mammary ephitelial cells genome was extracted from ENCODE [21] and a nucleotide alignment was performed with NCBI/ BLAST/ blastn suite.

### siRNA and Transfection

For knockdown with siRNA and hormone treatment experiments in absence of serum, UIII cells were cultured in FBS and, 24 hs later, media were replaced by white M199 with 10% dextran-coated charcoal- foetal bovine serum (DCC-FBS) and without antibiotics, in this conditions the cells were transfected. CDC2 siRNA (sc-29253, Santa Cruz Biotechnologies, California, USA), USF1 siRNA (sc-270501, Santa Cruz Biotechnologies, California, USA) or scramble siRNA (Negative control siRNA, Quiagen, Gene Glove) were used in 100 nM. Lipo 2000 (Lipofectamin 2000, Invitrogen) was used as the vehicle of transfection. Forty-eight hours later media were replaced by fresh M199 without serum and the cells were starved overnight. After one night in serum-free conditions, media were replaced by either vehicle or hormones.

### Western Blots

Protein samples were analyzed as described [17]. Quantification of blot intensities were performed with data obtained within a linear range of exposure (G:Box-Syngene). Details of these protocols in SI M&M.

### Chromatin Immunoprecipitation Experiments

ChIP experiments were performed as described [22]. UIII cells were seeded in 145 mm culture dishes and after hormonal treatments, chromatin was collected. The antibodies used for the immunoprecipitations were USF1 (Santa Cruz Bio. H-86), PR (Santa Cruz Bio. H-190) and normal rabbit IgG (Cell Signaling). The primers used for qPCR performed on immunoprecipitated (IP) and non-immunoprecipitated (input) DNA are detailed in Table S1 and S2. USF1 enrichment was expressed as percentage of input relative to T0 according to Comparative Ct method. Ct values were acquired with Bio Rad CFX Manager. PR enrichment was detailed in Figure 5C.

### Statistical Analysis

Analysis of variance followed by Tukey Multiple Comparison Test was used for statistical testing in all figures unless otherwise indicated. t-Test was performed to compare mRNA expression Fig. 2B and Fig. 5E, and cell number in Fig. 4E and Fig. 5F. Differences were considered significant if P<0.05. Statistical analysis was carried out with GraphPad Prim 4.0 (GraphPad Software Inc., La Jolla, CA, USA).

## Results

### R5020 Modulates Early Genes Expression in Stromal Endometrial UIII Cells

**Figure 1:**
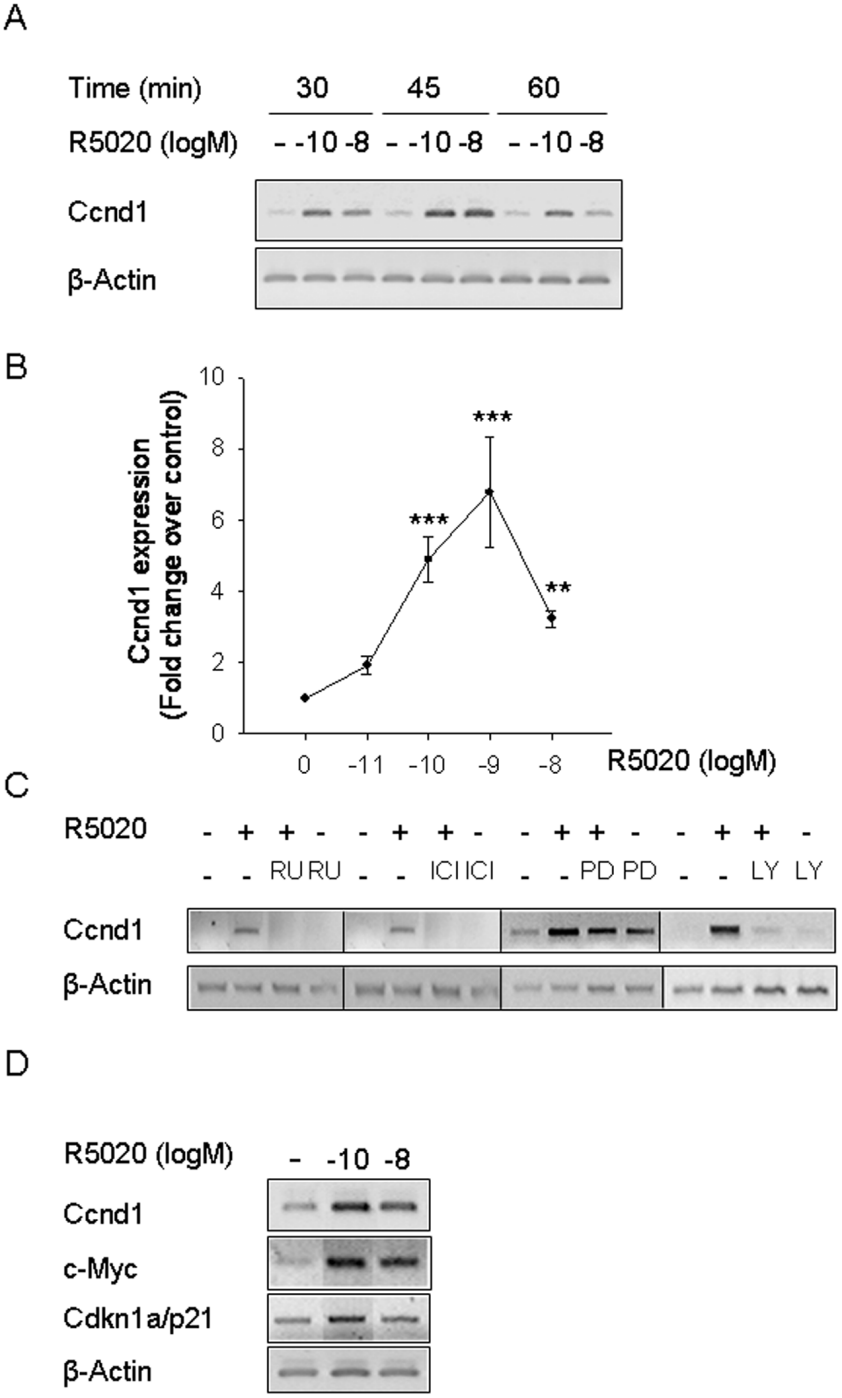
Low concentration of progestin increases *Ccnd1* transcript levels in UIII endometrial stromal cells. **A)** UIII cells were treated with vehicle (–), 10^−10^ M R5020 (−10) or 10^−8^ M R5020 (−8) during 30, 45 and 60 minutes in serum-free culture medium and total RNA was isolated and subjected to sq-PCR. Figure shows sybr green-stained gels of sq-PCR products for *Ccnd1* and *β-Actin* of a representative experiment selected from three independent experiments with similar results. **B)** UIII cells were treated with vehicle (0) or with increasing 10^−11^ M (–11) to 10^−8^ M (–8) concentrations of R5020 (R5020 (logM)) for 45 minutes. The graph represents the values for Ccnd1 fold change relative to β-Actin were divided by the vehicle-treated value (control). Data represent average ± SEM from 5 to 9 independent experiments. **P<0.01, ***P<0.001 v. vehicle. **C)** Antiprogestin RU486, antiestrogen ICI and inhibitors of ERK1-2 and AKT pathways effects on *Ccnd1* mRNA expression. Cells were pre-treated for 30 minutes with 10–^8^M RU486 (RU), 10–^7^ M ICI 182.780 (ICI), 50 µM PD 98.059 (PD) or 50 µM LY 294.002 (LY) followed by a 45 minute treatment with vehicle (–) or 10^−10^ M R5020 (+) as indicated. Figure shows sybr green-stained gels of sq-PCR products for Ccnd1 and *β-Actin* of a representative experiment selected from three independent experiments with similar results. **D)** *Ccnd1*, c-*Myc* and *Cdkn1a/p21* transcript expression was analysed in UIII cells treated as described in **B**.

We initiated the search for early downstream targets of the R5020 signaling pathway by exploring the optimal concentration and time point for gene expression profiling. Cyclin D1 (*Ccnd1*) was used as a well-known cell cycle regulator involved in progestin-dependent proliferation [23]–[24]. Two progestin concentrations were used: 10^−10^ M, known to activate the cytoplasmic initiated effects in UIII [17], and 10^−8^ M, usually associated to steroid transcriptional activation. Both concentrations transiently induced *Ccnd1* mRNA levels with a maximum at 45 minutes, but the lower concentration was more effective (Fig. 1A). When a larger range of R5020 concentrations was tested at 45 minutes, the optimal concentration was 10^−10^ to 10^−9^ M (Fig. 1B shows the statistical quantification of 3 experiments). Pre-treatment of the cells with the PR antagonist RU486 abolished the *Ccnd1* mRNA induction (Fig. 1C), indicating that it is mediated by the classical PR. The ER antagonist ICI 182780 had a similar effect revealing that *Ccnd1* regulation by R5020 requires ligand-free ER (Fig. 1C). Pre-treatment with PD 098.059 alone increased *Ccnd1* transcript levels (Fig. 1C) and diminished the increase in transcript levels induced by R5020, indicating that activation of ERK1-2 plays a complex role in controlling basal and hormone regulated *Ccnd1* expression. Instead, PI-3K/AKT inhibitor LY 294.002 (LY) blocked progestin induction of *Ccnd1*, suggesting that activated AKT is involved in *Ccnd1* progestin-dependent regulation (Fig. 1C). Another progesterone target gene *c-Myc* was also induced by 10^−10^ M R5020 in these conditions (Fig. 1D). *Cdkn1a/p21* mRNA was only slightly induced in early response to 10^−10^ M R5020 (Fig. 1D), but this gene is known to be regulated at later time points after progestin treatment [25].

### Transcription Factors and Cell Cycle Regulators are the Main Functional Categories of the Progestin-dependent Gene Network in Endometrial Stromal Cells

RNA analysis with oligonucleotide micro-arrays (Agilent 44 K arrays) from cells cultured for 45 minutes with vehicle or 10^−10^ M R5020 showed that 97 genes are significantly up-regulated (over 1.40-fold, B Rank ≥85B), and 341 genes are significantly down-regulated (more than −1.40 fold; B Rank ≥85B) (Tables S3 and S4, respectively; the fold change (FC) numbers represent the average from three biological replicates and a dye swap data set).

**Figure 2:**
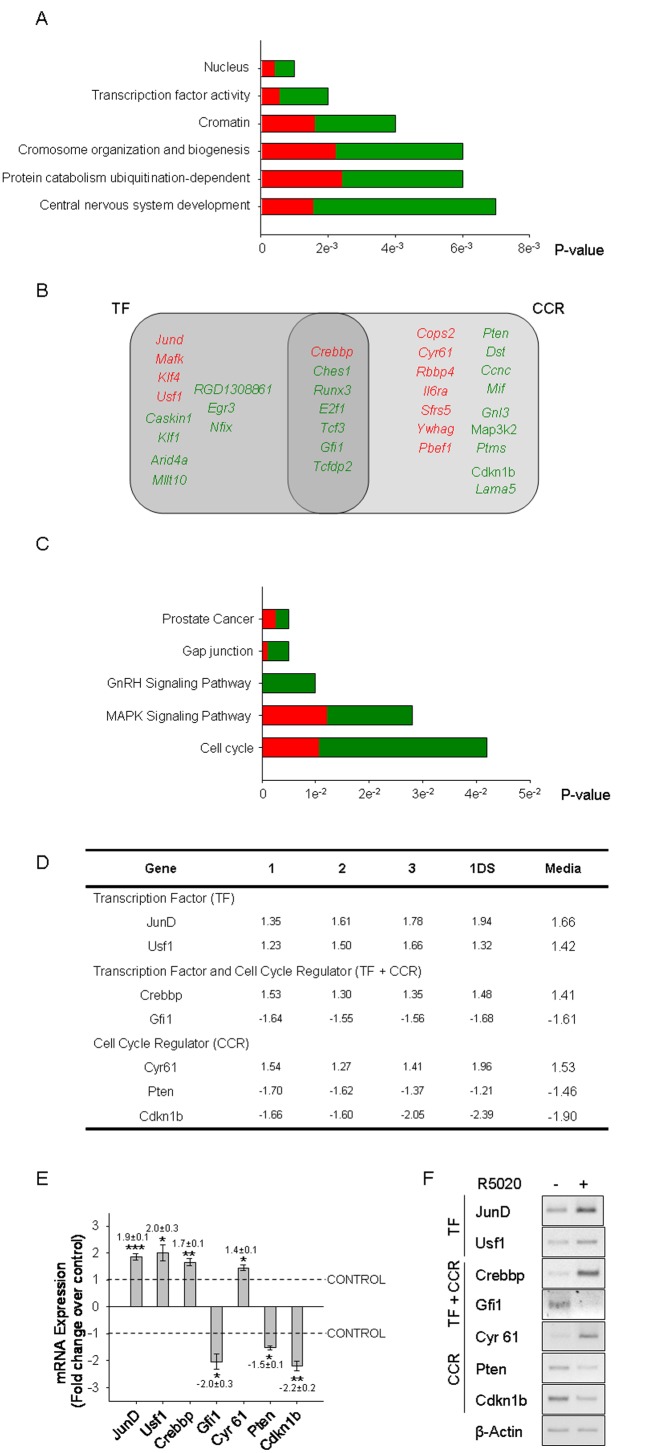
Transcription Factors and Cell Cycle Regulators are the main early progestin-regulated genes. **A)** The categories of over-represented Gene Ontology terms (GO terms) are shown by their decreasing p-values. The categories were identified by GOTM (Gene Ontology Tree Machine) software over the statistical regulated genes as indicated in supplementary Materials and Methods. Up-regulated genes percentages are shown in red, and down-regulated percentages are shown in green. **B)** Venn diagram shows the set of TF and CCR regulated by progestin. A GOTM search of cellular proliferation and cell cycle GO terms identified 23 genes, named cell cycle regulators (CCR) in the Figure. Seven of them were also categorized as transcription factors (TF) present in A. Up-regulated genes are shown in red, and down-regulated are shown in green. **C)** The signaling pathways associated to the differential gene expression pattern are shown by their decreasing p-value. Pathways identified by Pathway Express Software containing at least four progestin-dependent regulated genes included in a given Signalling Pathway (SP), with a p-value≤0.05. The percentage of up-regulated genes within a given signalling pathway is shown in red, and down-regulated genes are shown in green. Statistical details are described in M&M. **D)** The table shows individual fold changes of three independent biological samples (1,2,3) and one dye swap data set (1DS) analyzed by microarray, and the mean fold change of all 4 values (Media). Fold changes over vehicle treated cell values were calculated as described in SI M&M. **E)** q- real time PCR validation for *JunD*, *Usf1*, *Crebbp*, *Gfi1*, *Cyr61*, *Pten* and *Cdkn1b* mRNA relative to β-*Actin*. The figure shows media ± SEM from three to six independent experiments. *P<0.05, **P<0.01, ***P<0.001 v. vehicle treated cells. **F)** sq-PCR validation for transcription factors (TF) *JunD, Usf1*, transcription factors and cell cycle regulators (TF+CCR) *Crebbp, Gfi1, Cyr61*, and cell cycle regulators (CCR) *Pten, Cdkn1b* and *β-Actin*.

GOTM software (Gene Ontology Tree Machine) [26] analysis of the 438 progestin-regulated genes showed that the most significant differentially over-represented ontology terms were related mainly to transcription regulation: Cellular Component Categories, **nucleus** (44 genes, with P = 0.001) and **chromatin** (5 genes with P = 0.004); Functional Component Categories, **transcription factor activity** (18 genes, 5 of which were up-regulated, *JunD*, *Mafk*, *Klf4*, *Usf1*, *Crebbp*, and 13 were down-regulated *RGD1308861*, *Egr3*, *Nfix*, *Ches1*, *Runx3*, *E2f1*, *Tcf3*, *Caskin1*, *Klf1*, *Gfi1*, *Tcfdp2*, *Arid4a*, *Mllt10,* with P = 0.002) (Fig. 2B, TF), **chromosome organization and biogenesis** (8 genes, 3 of which were up-regulated, *Rbbp4*, *Crebbp*, *Pdcd8*, and the remaining 5 were down-regulated, *Smarcc1*, *Setdb1*, *Tnks1bp1*, *Tcf3*, *Klf1*, with P = 0.006), **ubiquitination-dependent protein catabolism** (2 up-regulated genes, *Ube3a*, *Ube2n*, and 3 down-regulated genes, *Ate1*, *Arih2*, *Usp7*, with P = 0.006), and **central nervous system development** (2 up-regulated genes, *Klf4*, *Ube3a*, and 7 down-regulated genes, *B3gnt5*, *Odz2*, *Pitx3*, *E2f1*, *Atrx*, *Sept4*, *Pitpnm1*, with P = 0.007) (Fig. 2A).

The search for the ontology terms Cellular Proliferation and Cell Cycle yielded 23 genes (Fig. 2B, CCR-Cell Cycle Regulators). Seven of them are transcription factors (*Crebbp*, *Ches1, Runx3 E2f1, Tcf3, Gfi1* and *Tcfd2*) and 3 are chromatin modifiers (*Rbbp4*, *Tcf3* and *Crebbp*). Eight genes were up-regulated and 15 were down-regulated, of which 7 were inhibitory functions (Fig. 2B). *Ches1, Tcf3, Ccnc and Gfi1* are transcriptional repressors, *Pten* and *Runx3* are tumor suppressors, and *Cdkn1b* is a cell cycle inhibitor. These findings suggest that the progestin-dependent proliferation could be partly achieved via inhibition of repressors and partly via regulation of immediate early genes associated to the transcriptional control of cell cycle regulatory molecules.

The Pathway Express Onto-Tool [27] identified seven genes (*Cacng2, Jund, Mras, Pdgfrb, Pla2g2c, Ppp3r1 Sos1*) encompassed in the MAPK (Mitogen-activated protein kinases) signaling pathway, confirming the significance of MAPK signalling in the early response to progestin in UIII cells (Fig. 2C).

The expression of transcription factors and cell cycle regulators found by microarrays was validated using q-PCR. The mean fold changes obtained by microarrays and q-PCR are shown in Fig. 2D and 2E respectively, and confirm the validity of the microarray data. The results of sq-PCRs stained with sybr green are shown as well (Fig. 2F).

### Role of Hormone Receptors and Kinases on Progestin Gene Regulation

**Figure 3:**
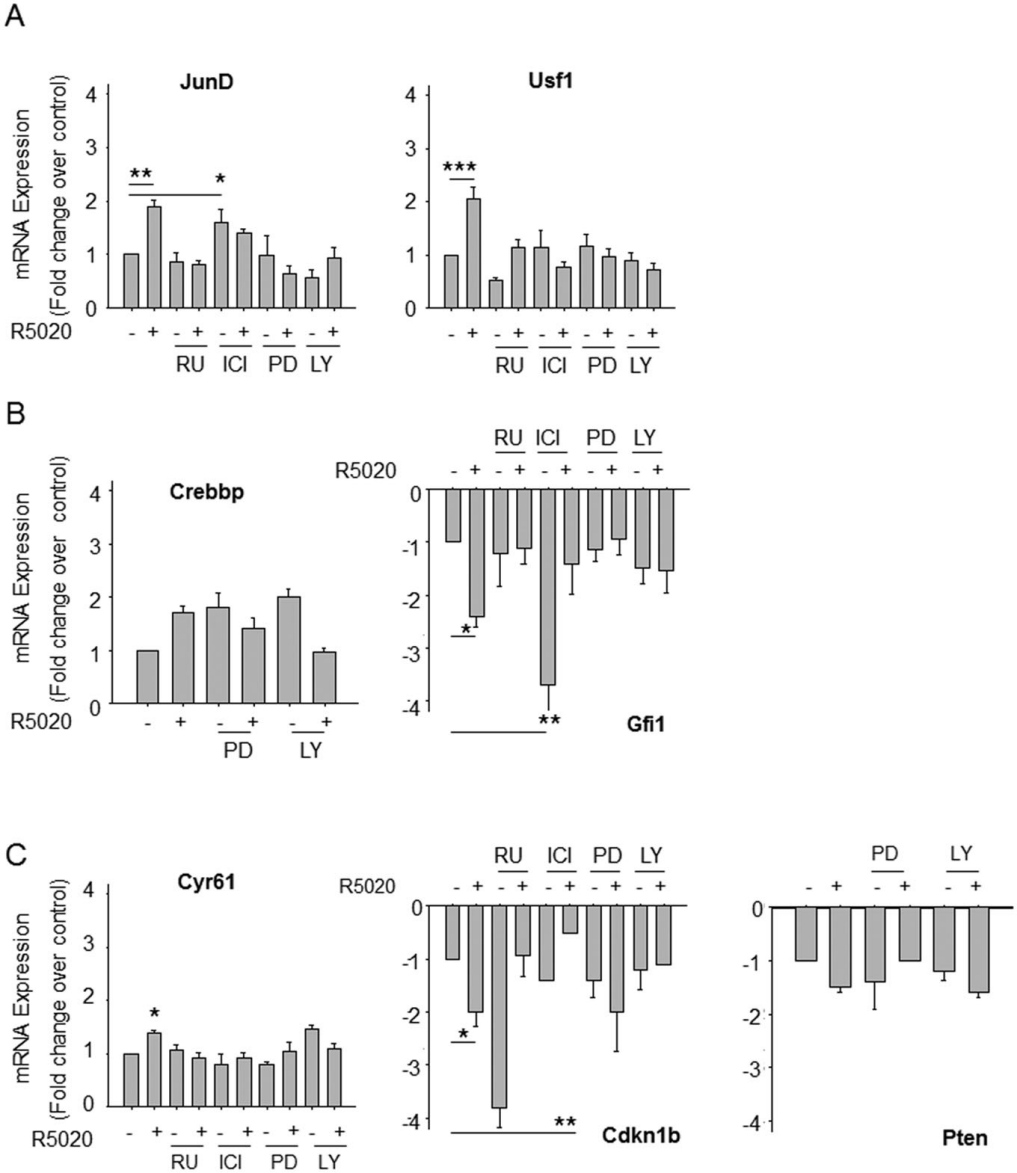
PR, ER, ERK1-2 and AKT activation on progestin-regulated transcription factors and cell cycle regulators mRNAs. UIII cells were pre-treated for 30 minutes with 10^−8^ M RU486 (RU), 10^−7^ M ICI 182.780 (ICI), 50 µM PD 98.059 (PD) or 50 µM LY 294.002 (LY) followed by a 45 minute treatment with vehicle (–) or 10^−10^ M R5020 (+) when indicated. **A)** q-PCR of mRNAs from *JunD* and *Usf1* relative to *β-Actin* mRNA. **B)** q-PCR of *Crebbp* and *Gfi1* mRNA relative to *β-Actin* mRNA. **C)** q-PCR of *Cyr61*, *Cdkn1b* and *Pten* mRNA relative to *β-Actin* mRNA. The figures show media ± SEM from three independent experiments. *P<0.05, **P<0.01, ***P<0.001 vs vehicle treated cells.

We used hormone receptor antagonists and kinase specific inhibitors to study the involvement of PR (RU), ER (ICI), ERK1-2 (PD) and AKT (LY) on progestin regulation of the validated target genes including *JunD*, *Usf1*, *Crebbp*, *Gfi1*, *Cyr61*, *Pten* and *Cdkn1b*. Figure 3 shows the values of changes in gene expression of the tested genes in cells treated with 10^−10^ M R5020 for 45 min after preincubation for 30 min with vehicle or with RU486, ICI182780, PD98059 and LY294002. PR antagonist RU486 blocked up-regulation of *JunD*, *Cyr61* and *Usf1* (Fig. 3A and 3C), as well as down-regulation of *Gfi1* and *Cdkn1b* (Fig. 3B and 3C). In absence of progestin, the antagonist treatment down-regulated *Gfi1* transcript expression, indicating that basal *Gfi1* transcription requires ligand-free PR (Fig. 3B). These results show that progestin regulates *JunD*, *Cyr61*, *Usf1*, *Gfi1* and *Cdkn1b* through classic PR.

ER antagonist ICI 182780 revealed that *Usf1*, *Gfi1*, *Cyr61*, and *Cdkn1b* regulation by R5020 requires ligand-free ER (Fig. 3A, 3B and 3C). *JunD* mRNA expression level in the presence of ICI alone was similar to its expression in the presence of ICI+R5020 and higher than in control conditions (Fig. 3A). However, the level of *JunD* mRNA in presence of both R5020 and ICI was lower than with R5020 alone. Additionally, ICI treatment compromised the response of *JunD* and *Cdkn1b* genes to R5020 due to a differential effect on the basal activity of the two genes: whereas it increased basal activity of *JunD*, it decreased basal activity of *Cdkn1B*. We conclude that, in addition to PR, ER is partially involved in the induction of *JunD* by progestin (Fig. 3A). Pre-treatment with ICI blocks the progestin down-regulation of *Gfi1* and *Cdkn1b*, suggesting that the ligand-free ER is required for progestin gene repression.

Pre-treatment with PD 098.059 abolished R5020 induction of *JunD*, *Usf1*, and *Cyr61* (Fig. 3A and 3C), as well as down-regulation of *Gfi1* and *Cdkn1b* (Fig. 3B and 3C).

PI-3K/AKT inhibitor LY 294.002 (LY) precludes progestin induction of *JunD*, *Usf1* and *Cyr61* as well as repression of *Gfi1* and *Cdkn1b* (Fig. 3A, 3B and 3C). In the presence of LY, R5020 reduced *Pten* mRNA levels to a lower extent than in its absence. Although the statistical significance of this finding remains to be established, the data suggest that progestin-dependent inhibition of *Pten* transcript expression requires ERK1-2 activation and is independent of AKT activation.

Although individual genes show more complex behaviours, such as *Gfi1* in response to RU and *Cdkn1b* in response to ICI, the inhibitory effects of RU, ICI, PD and LY suggest that regulation by progestin R5020 of these genes needs classical PR, ER, ERK1-2 and/or AKT activation. Thus at least two different signaling pathways are involved in the short-time progestin control of the expression of these transcription factors and cell cycle regulators.

Since the effects of ICI indicate a signaling pathway that involves ER activation in the absence of estrogens, we have tested the effect of estrogens on the expression of validated R5020-regulated genes. Except in the case of Gfi1 repression, estradiol (10^−8^ M) did not show the same regulation pattern as observed with R5020 (Fig. S1), suggesting that the outcome of activation of the PR-ERbeta pathway does not depend on estradiol as a ligand.

In summary, these results show that progestin-dependent regulation is a consequence of ERK1-2 and/or AKT activation that requires ligand-free ER. Thus at least two different kinase signaling pathways are involved in the short-time progestin control of the expression of these transcription factors and cell cycle regulators.

### Downstream Targets of Progestin-regulated Transcription Factors *Usf1* and *Crebbp*

We next investigated the involvement of downstream target genes of regulated transcription factors in progestin-dependent proliferation. *Cdc2* and *cyclin b1* (*Ccnb1*) have been described as USF1 target genes [28]–[29], while *p21* (*Cdkn1a*) and *c-My*c are regulated by GFI1[30], and *Cdc6* and *cyclin E* (*CcnE*) are targets of CREBBP [31].

**Figure 4:**
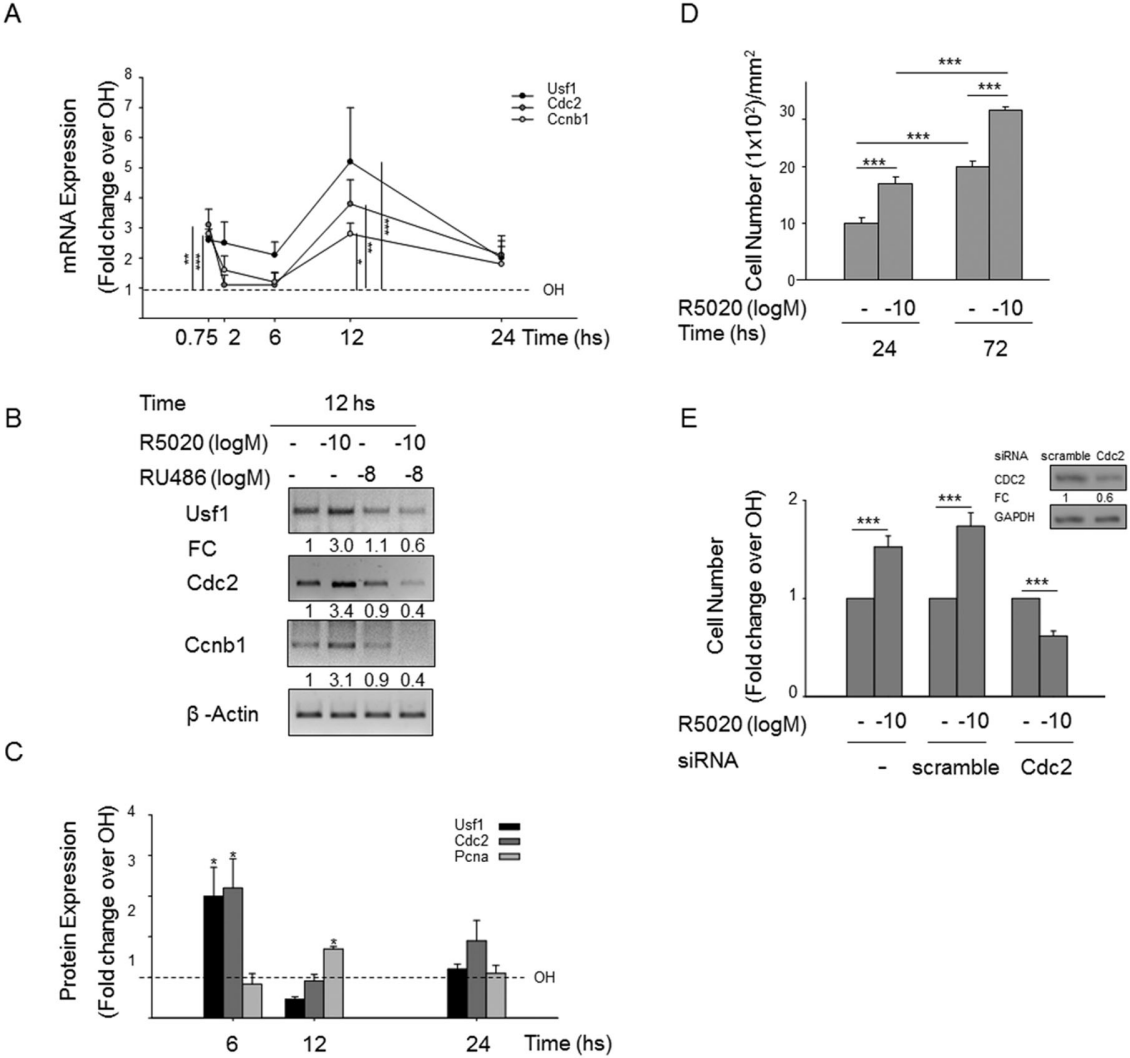
CDC2, a USF1 target, is responsible for progestin-induced UIII cell growth. **A)** UIII cells were treated at 45 minutes, 2, 6, 12 and 24 h with 10^−10^ M R5020 as indicated in Fig. 1A. The values for mRNAs fold change of *Usf1, Cdc2, Ccnb1* relative to *β-Actin* were divided by the vehicle-treated value for each time point tested. Data represent average ± SEM from 3 to 5 independent experiments. *P<0.05, **P<0.01, ***P<0.001 v. vehicle. **B)** shows representative products of sq-PCR of these genes in 30 minutes RU486 pre-treated cells from three independent experiments with similar results. **C)** shows USF1, CDC2 and PCNA protein expression in which fold change relative to ERK2 was divided by the vehicle-treated value at 6, 12 and 24 h progestin treatment. Data represent average ± SEM from 3 independent experiments. *P<0.05, **P<0.01, ***P<0.001 v. vehicle. **D)** Number of cells after 24 and 72 h of vehicle (−) and 10^−10^ M progestin treatments in UIII cells were treated as indicated in Fig. 1B. ***P<0.001. **E)** Number of cells transfected without siRNA, with scramble siRNA and with *Cdc2* siRNA 24 h before treatment with vehicle and progestin. ***P<0.001. Lines in both figures indicate statistical comparison; standard deviation is indicated. **Inset E)** Western blots for CDC2 and GAPDH of cell transfected with *Cdc2* and scramble siRNAs.

Time-course experiments of response to R5020 showed that the levels of mRNA for *Usf1* increased at 45 min, decreased slightly at 2 and 6 hours and reached a maximum at 12 hours (Fig. 4A). This pattern gathers strength for the *Cdc2* mRNA levels that were up-regulated at 45 min, returned to control levels at 2 and 6 hours, and increased again at 12–24 hours (Fig. 4A). *Ccnb1* mRNA was also transiently up-regulated at 45 min and at 12 hours (Fig. 4A). These effects are all mediated by PR as they were blocked by the antiprogestin RU486 at 12 hours treatment (Fig. 4B).

Through western blot, we analyzed the protein levels of USF1, its target CDC2/CDK1, the S-phase marker PCNA, and total ERK2 in cells treated with vehicle or 10^−10^ M R5020 during 6, 12 and 24 hours (Fig. 4C). USF1 and CDC2 were increased at 6 and 24 hours, and showed basal levels at 12 hours (Fig. 4C). On the other hand, PCNA increased only at 12 hours, suggesting that DNA synthesis occurs as a consequence of the initial rise in USF1 and in its target CDC2 at 6 hours (Fig. 4C).

In addition to USF1 targets, we also looked at CREBBP target CcnE. Although significant *Crebbp* mRNA induction by progestin was not consistently observed, the mRNA levels of *CcnE*, a target gene of CREBBP (Fig. S2A), increased gradually from 45 min to 12 hours in response to progestin treatment. *CcnE* progestin dependent induction after a 12 hours treatment was abolished by RU486 pretreatment (Fig. S2B), indicating the need for the classic PR.

### USF1-induced CDC2 is Required for Progestin-dependent Proliferation

After 24 hours of progestin treatment, the cell number increased 2 fold (Fig. 4D). A 60 percent depletion of CDC2 with specific siRNA (Fig. 4E insert) precluded progestin dependent cell proliferation while cell proliferation was maintained in cells transfected with scramble siRNA (Fig. 4E), demonstrating that CDC2 mediates progestin-dependent proliferation.

**Figure 5:**
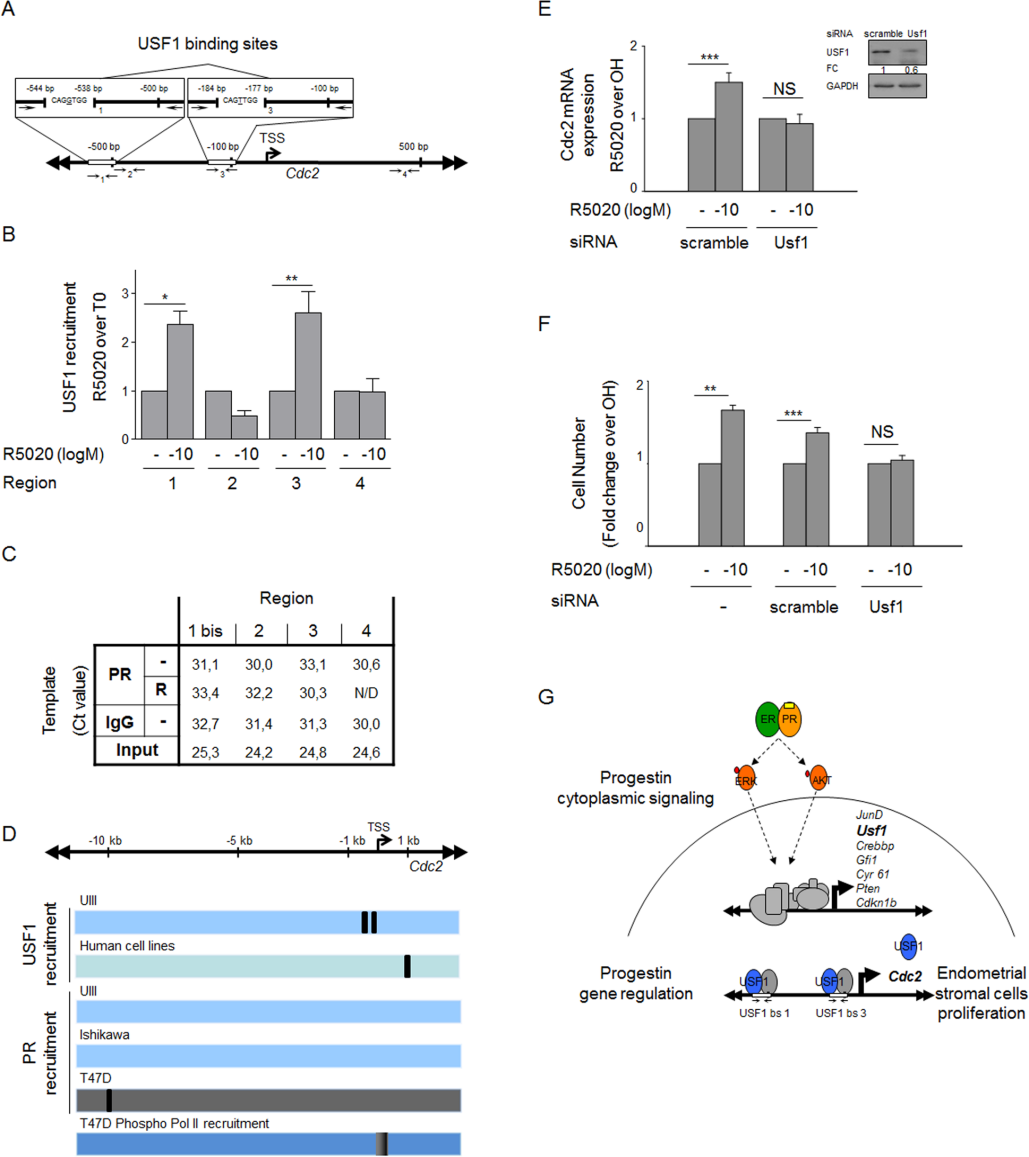
Progestin directs USF1 binding to *Cdc2* promoter. **A)** *Cdc2* proximal promoter (–0.75 Kb to +0.5 Kb) contains two specific heptamers binding sites for USFs and two nearby unrelated regions. **B)** USF1 is recruited to the *Cdc2* proximal promoter sequence. UIII cells were treated with 10–10 M R5020 for 30 min and subjected to ChIP with IgG as control, or a USF1 antibody and the purified DNA was used for q-PCR. The values represent average ±β SEM fold changes relative to inputs over T0 from 3 independent experiments for each of the four regions (1–4) shown in the upper *Cdc2* promoter scheme. *P<0.05, **P<0.01 v. vehicle. **C)** PR is not recruited to the *Cdc2* proximal promoter sequence. After performing PR ChIP in untreated (−) and R5020 treated cells (R), we analyzed the recruitment to *Cdc2* proximal promoter sequence by qPCR using 4 pairs of primers, 3 of which were used for USF1 ChIP analysis (Regions 2, 3, and 4) and a pair (1 bis), which covers the corresponding region (Figure 5C). Ct: Crossing threshold, N/D: not detected. **D)** USF1, PR and phospho Pol II recruitment to *Cdc2* promoter (−10 Kb to +1 Kb) in UIII rat endometrial cells, in human cell lines (ENCODE: A549 epithelial cell line derived from a lung carcinoma tissue, H1-hESC embryonic stem cells, HepG2 hepatocellular carcinoma), Ishikawa human endometrial cells [La Greca et al, unpublished data], and T47D epithelial mammary cells [30]. **E)** USF1 mediates progestin regulation of Cdc2. The graph shows *Cdc2* mRNA expression determined by q-PCR after 12 h of treatment with vehicle and progestin of cells transfected with scramble siRNA and with *Usf1* siRNA 48 h before. ***P<0.001, NS: Non Significant. Lines indicate statistical comparison; standard deviation is indicated. **Inset E)** Picture shows western blots for USF1 and GAPDH of cells transfected with *Usf1* and scramble siRNAs. **F)** Number of cells transfected without siRNA, with scramble siRNA and with *Usf1* siRNA 24 h before treatment with vehicle and progestin. **P<0.01, ***P<0.001, NS: not significant. Lines indicate statistical comparison; standard deviation is indicated. **G)** Model depicting PR-ER, ERK and AKT activation necessary for CDC2 to mediate Progestin initiated endometrial stromal cell proliferation independently of PR binding to chromatin. Progestin binding to PR from a PR-ER preformed complex activates ERK and AKT at the cytoplasm. The progestin cytoplasmic signaling differentially regulates early gene expression, mainly of cell cycle regulators and transcription factors. The transcription factor USF1 is recruited to *Cdc2* proximal promoter, and *Cdc2* induction under hormone treatment is required for progestin-dependent endometrial stromal cells proliferation.

To explore the mechanism of USF1 transcription factor regulation of *Cdc2* expression, we performed ChIPs experiments over a region of *Cdc2* promoter (see *Cdc2* promoter pattern at Figure 5A), which contains two specific nucleotide heptamers known to function as binding sites for USFs and to be highly conserved in different species [28]. This ChIP showed that after 30 min of treatment with R5020 USF1 binds to both *Cdc2* promoter sequences (Figure 5B, regions 1 and 3) while two other nearby regions were negative for USF1 binding (Figure 5B, regions 2 and 4). These results are consistent with binding of USF1 to its target *Cdc2* mediating the regulated expression of this cell cycle kinase in UIII cells.

We have shown that endogenous PR is not able to induce transcription of an exogenous PRE construction in UIII cells [17]. Therefore, we did not expect to find PR binding to DNA target sequences. Nevertheless, to exclude PR direct interaction with *Cdc2* promoter, prior to hormone treatment (T0) and 60 min after addition of 10^−10^ M R5020 (R60), we tested PR binding to *Cdc2* proximal promoter, −0.75 Kb to +0.5 Kb relative to the transcription start site (TSS) using 3 of the USF1 ChIP pairs of primers (regions 2, 3 and 4 in Fig. 5B) and an extra pair which partially overlaps with region 1 (region 1 bis in Figure 5C). The results confirmed the absence of PR binding to *Cdc2 *DNA proximal promoter sequence (Fig. 5C) although we do not exclude binding to other distant regions of the gene. PR is recruited on a distal position (−10.9 Kb to −10.7 Kb) of *Cdc2* promoter in T47D human mammary ephitelial cells under progestin treatment [21] and this region contain several potential PREs (TGTYCY), but this region is not conserved in mouse or rat genomes (NCBI/ BLAST/ blastn suite).

To compare *Cdc2* regulation by USF1 and PR, we analyzed USF1 recruitment in ENCODE human cell lines (http://genome.ucsc.edu/) [32] and PR recruitment in human Ishikawa endometrial cells (La Greca A et al., unpublished data) and T47D cells [21]. The USF1 element in *Cdc2* described in A549 epithelial cell line derived from a lung carcinoma tissue-, H1-hESC embryonic stem cells and HepG2 hepatocellular carcinoma was in the region +1044 to +1300 from the TSS, differently positioned from what we found for USF1 recruitment (Fig. 5D). PR recruitment in *Cdc2* of human Ishikawa endometrial cells was negative (La Greca et al., unpublished data), while it was positive in T47D cells [21], hinting at possible tissue specificity.

Progestin-dependent regulation of *Cdc2* is evident from the fact that after 12 hours of progestin treatment, the *Cdc2* transcript expression increased around four times over vehicle treated cells (Fig. 4A). To confirm that progestin regulation of *Cdc2* was mediated by USF1, we knocked down USF1 and measured *Cdc2* mRNA after 12 hours of progestin and vehicle treatment. A 60 percent depletion of USF1 with specific siRNA (see Western blot in Fig. 5E insert) abolished progestin-dependent *Cdc2* transcript upregulation, while *Cdc2* mRNA expression was not modified in cells transfected with scramble siRNA (Fig. 5E). This finding demonstrates that *Cdc2* direct transcriptional regulation is mediated by progestin-dependent USF1. Also, USF1 specific siRNA abrogated progestin-dependent proliferation (Fig. 5F). Taken together, these results point to Cdc2 as a possible cell cycle target of early transcription factor USF1 whose expression is selectively induced by progestin via the interaction of PR-ERbeta and ERK and AKT activation independently of PR binding to genomic targets (Fig. 5G).

## Discussion

In this study we explored the possibility that steroid hormones can regulate gene expression via activation of kinase signalling pathways without requiring a direct interaction of their receptors with the target genes in chromatin. As we have shown previously, UIII cells are a good model system to study ¨cytoplasmic initiated effects” of low concentration of progestin in the presence of PR and ERb but not ERa and without transcriptional activity of both endogenous receptors [17]. Here we identify transcription factors as downstream molecular targets of progestin activation of ERK1-2 and AKT involved in proliferation of UIII cells and specifically explore CDC2 –a USF1 transcription factor target- involved in proliferation.

We first analyzed the expression of cyclinD1, the regulatory component of the complex CyclinD1-CDK4 that, together with cyclinE-CDK2, promotes S phase entry through the phosphorilation of pRb [33]. Even though there are no progesterone response elements (PRE) on *Ccnd1*, progestin regulates its transcription through a proximal promoter c-Ets-2 binding site [34]–[35]. We found a rapid and transient induction of *Ccnd1* transcripts in progestin treated UIII cells mediated by classical PR. Unexpectedly, optimal transcriptional activation was observed in response to low progestin concentrations, 10^−10^ M and 10^−9^ M R5020. This observation is consistent with our previous results showing that the optimal concentration of progestin inducing UIII cell proliferation as well as ERK1-2 and Akt activation is in the subnanomolar range, and suggests that *Ccnd1* is a mediator of the proliferative response. Therefore we used a low concentration of progestin (R5020 10^−10^ M) and a short time treatment (45 minutes) to identify the global set of early target genes involved in the initiation of progestin-dependent proliferation in endometrial cells.

Using oligonucleotide microarrays, we identified 438 regulated genes, of which 78 percent were significantly down-regulated, suggesting that repression could be a relevant molecular mechanism by which progesterone regulates stromal proliferation in endometrium. The over-represented ontology terms revealed that progestin downstream genes are mainly involved in the regulation of transcription, notably transcription factors, steroid receptor co-regulators and chromatin remodelling molecules/regulators/modifiers/. We detected a set of 23 genes encompassed in the Cellular Proliferation and Cell Cycle ontology terms and found that 9 of them were also present in the overrepresented set of transcription factors and chromatin remodelling genes. We validated the expression of genes described as transcription factors (*JunD*, *Usf1*), as transcription factors and cell cycle regulators (*Crebbp* and *Gfi1*), and as cell cycle regulators (*Cyr61*, *Pten* and *Cdkn1b*).

*Usf1* (up-regulated 1.42-fold) encodes a ubiquitous transcription factor that regulates gene networks involved in stress and immune response, cell cycle and cell proliferation. USF transcription factors have been shown to be targets of ERK1-2 in epidermal keratinocytes [36]. USF1 controls cell proliferation by targeting cell cycle genes such as *p53* [37], *Cdc2* [28] and *cyclin b1* [29]. CDC2 or cyclin dependent kinase1 (CDK1) forms a complex with CyclinB1, whose activation by phosphorylation promotes the entry in the mitotic phase of the cell cycle [38]. USF1 specifically mediates transcriptional activation of *Ccnb1* (Cyclin B1) just before and during mitosis in HeLa cells [29].

We found that *Usf1* and its targets *Cdc2* and *Ccnb1* were transiently induced at 45 min and at a later time (12 hours). USF1 and CDC2 proteins also showed progestin-dependent regulation. CDC2 hormone-dependent regulation preceded PCNA increase, supporting recent evidence that CDC2 regulates G1 progress and G1-S phase transition [39]. It is to be noted that the reduction of CDC2 by means of siRNA UIII cells did not respond to progestin effects.

To test USF1 regulation of *Cdc2* at a transcriptional level we performed ChIP and tested USF1 recruitment over *Cdc2* proximal promoter. Our results show a USF1 progestin-dependent recruitment to two specific heptamers in *Cdc2* promoter region, which were described as USF binding sites [28]. The genomic position of these bindings was different from the one described for several human cell lines in ENCONDE project (http://genome.ucsc.edu/). We confirmed the absence of PR binding to *Cdc2 *DNA proximal promoter sequence in accordance with the absence of PR binding in Chip seq experiments performed in Ishikawa cells treated with R5020 (data not shown) and in T47D cells treated with R5020 [21]. This encourages the study of *Cdc2* gene expression regulation mediated by cytoplasmic PR pathway. A functional approach using siRNA showed that depletion of USF1 abolished *Cdc2* progestin-dependent transcriptional regulation and progestin-dependet proliferation, confirming that USF1 mediates the progestin transcriptional regulation of *Cdc2* and R5020 induced proliferation.

To explore the repertoire of the described transcription factors binding sites to the complete set of TF regulated by R5020 we performed an in silico search using TRANSFAC software (www.gene-regulation.com) over the set of 32 progestin regulated genes shown in Fig. 2D. This analysis resulted in the absence of PR elements (PRE), and in the presence of steroid hormone response elements (SHRE) in 3 genes, of USF1 elements in 15 genes, of ELK1 elements in 14 genes and of other non-SHRE in the remaining 13 genes. This pattern is consistent with non-direct binding of PR to this set of kinase regulated genes.

Our finding suggests that the progestin regulated interaction between USF1 and its target *Cdc2* could regulate the expression of this cell cycle kinase in UIII cells in the absence of genomic PR binding.

Our present results identify for the first time in endometrial stromal cells a set of early target genes of progestin-activated classical PR via ERb and protein kinases. Progestin regulation of *JunD*, *Usf1*, *Gfi*, *Pten*, *Cdkn1b* and *Cyr61* required ERK1-2 or AKT activation and ligand-free ERb, while regulation of *Ccnd1* was mediated by activation of AKT but not ERK1-2. This study suggests that the downstream targets of cytoplasmic kinases activated by PR in UIII cells are predominantly early induced transcription factors and repressors or inhibitors of cell proliferation that are down-regulated by progestins, as well as activators of cell cycle regulators. One of them, CDC2, is up-regulated by USF1 and required for progestin-induced proliferation in a pathway that does not bind PR to *Cdc2* proximal promoter.

## Supporting Information

Figure S1 **Estradiol effects on validated Transcription Factors and Cell Cycle Regulators.** UIII cells were treated as described in Figure 1B and treated with vehicle (OH), R5020 10^−10^ M (R10) or Estradiol 10^−8^ M (E8) for 45 minutes in a serum-free culture medium. Graphs show *JunD, Usf1, Cyr61, Cdkn1b* mRNAs expression determined by q-PCR. *Gfi1, Ccnd1* and *c-Myc* mRNAs expression was analyzed by sq-PCR and representative electrophoresis gels stained with sybr-green are shown in the insets. In all cases values for gene fold change relative to *β-Actin* were divided by the vehicle-treated value. Data represent average ± SEM from 3–5 independent experiments. *P<0.05, **P<0.01, ***P<0.001.
(TIF)

Figure S2 **Progestin regulation of** ***Crebbp*** **transcription factor targets.** UIII cells were treated as indicated in Fig. 1D. The values for mRNAs fold change relative to *β-Actin* were divided by the vehicle-treated value for each time point tested. **A)** *Crebbp, CcnE* and *β-Actin* at 45 minutes, 2, 6, 12 and 24 h of 10^−10^ M R5020. Data represent average ± SEM from 3 to 5 independent experiments. **P<0.01 vs vehicle. **B)** representative products of sq-PCR of these genes in 30 minutes RU486 pre-treated cells from three independent experiments with similar results.
(TIF)

Table S1 **PCR primer sequences designed by OLIGO Primer Analysis Software (Molecular Biology Insights, Inc.).**
(DOC)

Table S2 **PCR primers position relative to Cdc2 Transcription Start Site (TSS).** Primers Ubs 1 and 3 correspond to region 1 and 3 respectively, while primers nUbs 2 and 4 correspond to regions 2 and 4 respectively in figure 5C. Primers Ubs 1 bis are located just upstream of the Ubs 1 pair and cover a region which partially overlaps with region 1, namely 1 bis.
(DOC)

Table S3 **Progestin-dependent up-regulated gene expression pattern.** The table shows individual fold changes of up-regulated genes after 45 min treatment with R5020 10^−10 ^M related to vehicle. Data were taken from three independent samples (E1, E2, E3) and one dye swap experiment (1DS) analyzed by microarray and expressed by mean fold change of all 4 values (FC). Colour scale for up (red), non (black) and down (green) regulated genes is shown.
(DOC)

Table S4 **Progestin-dependent down-regulated gene expression pattern.** The table shows individual fold changes of statistical down-regulated genes after 45 min treatment with R5020 10^−10 ^M related to vehicle. Down (green) regulated genes are ordered by increasing mean fold change. Data shown as indicated in Table S3.
(DOC)
