# Correction: CDC2 Mediates Progestin Initiated Endometrial Stromal Cell Proliferation: A PR Signaling to Gene Expression Independently of Its Binding to Chromatin

**DOI:** 10.1371/journal.pone.0321979

**Published:** 2025-04-03

**Authors:** Griselda Vallejo, Alejandro D. La Greca, Inti C. Tarifa-Reischle, Ana C. Mestre-Citrinovitz, Cecilia Ballaré, Miguel Beato, Patricia Saragüeta

After publication of this article [1], concerns were raised about results presented in Fig 1. Specifically:

**Fig 1:**
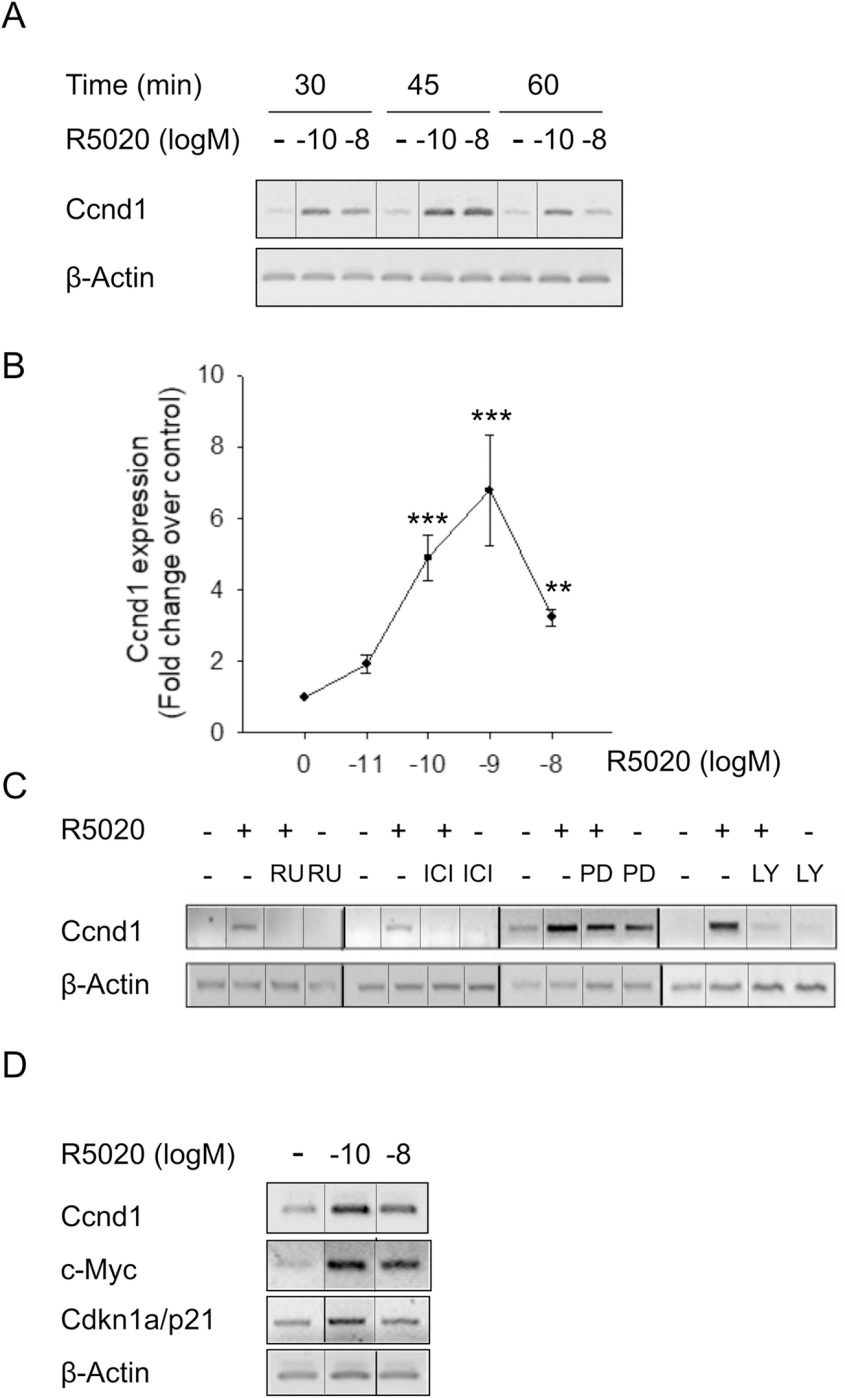
Low concentration of progestin increases *Ccnd1* transcript levels in UIII endometrial stromal cells. A) UIII cells were treated with vehicle (-), 10^-10^M R5020 (-10) or 10^-8^M R5020 (-8) during 30, 45 and 60 minutes in serum-free culture medium and total RNA was isolated and subjected to sq-PCR. Figure shows sybr green-stained gels of sq-PCR products for *Ccnd1* and *β-Actin* of a representative experiment selected from three independent experiments with similar results. Non-adjacent bands from the same gel are indicated with slim solid black lines. B) UIII cells were treated with vehicle (0) or with increasing 10^-11^M (-11) to 10^-8^M (-8) concentrations of R5020 (R5020 (logM)) for 45 minutes. The graph represents the values for Ccnd1 fold change relative to β-Actin were divided by the vehicle-treated value (control). Data represent average ±SEM from 4 to 9 independent experiments. ** P<0.01, *** P<0.001 v. vehicle. C) Antiprogestin RU486, antiestrogen ICI and inhibitors of ERK1-2 and AKT pathways effects on *Ccnd1* mRNA expression. Cells were pre-treated for 30 minutes with 10-8 **M** RU486 (RU), 10-7 **M** ICI 182.780 (ICI), 50µM PD 98.059 (PD) or 50µM LY 294.002 (LY) followed by a 45 minute treatment with vehicle (-) or 10^-10^ **M** R5020 (+) as indicated. Figure shows sybr green-stained gels of sq-PCR products for Ccnd1 and *β-Actin* of a representative experiment selected from three independent experiments with similar results. Non-adjacent bands from the same gel are indicated with slim solid black lines. D) *Ccnd1*, c-*Myc* and *Cdkn1a/p21* transcript expression was analysed in UIII cells treated as described in B. Non-adjacent bands from the same gel are indicated with slim solid black lines.

- In Fig 1C, lanes 1-4 of the CCnd1 and β-Actin panels appear similar to lanes 5-8 of the same CCnd1 and β-Actin panels
- There appear to be vertical discontinuities in the Ccnd1 panel of Fig 1C between lanes 1 and 2, and lanes 5 and 6.
- In Fig 1D there appear to be vertical discontinuities on either side of lane 2 in the c-Myc and Cdkn1a/p21 panels.

The corresponding author stated that lanes 5–8 of the CCnd1 and β-Actin panels in Fig 1C are incorrect, and that this was not observed prior to publication as the RU antagonist effect over R5020 is the same as the ICI antagonist effect over R5020. The corresponding author further clarified that the CCnd1 and β-Actin panels in Fig 1C represent the same experimental samples run on different agarose gels, that the same cDNA template for each experimental sample was used for beta actin PCR and Ccnd1 PCR, and that the PCR products were then run on different agarose gels.

The corresponding author also stated that the vertical discontinuities in Figs 1, 2 and 4 are due to the original experiments being carried out alongside additional treatments not included in [1]. A correct version of Fig 1 is provided here where non-adjacent bands from the same gel are indicated with solid black lines in Figs 1A, C and D, and where lanes 5–8 of the CCnd1 and β-Actin panels in Fig 1C have been updated. Updated versions of Figs 2 and 4 are provided here where non-adjacent bands from the same gel are indicated with solid black lines in Figs 2H and 4B. The corresponding author stated that they did not generate panels from lanes from different gels.

**Fig 2:**
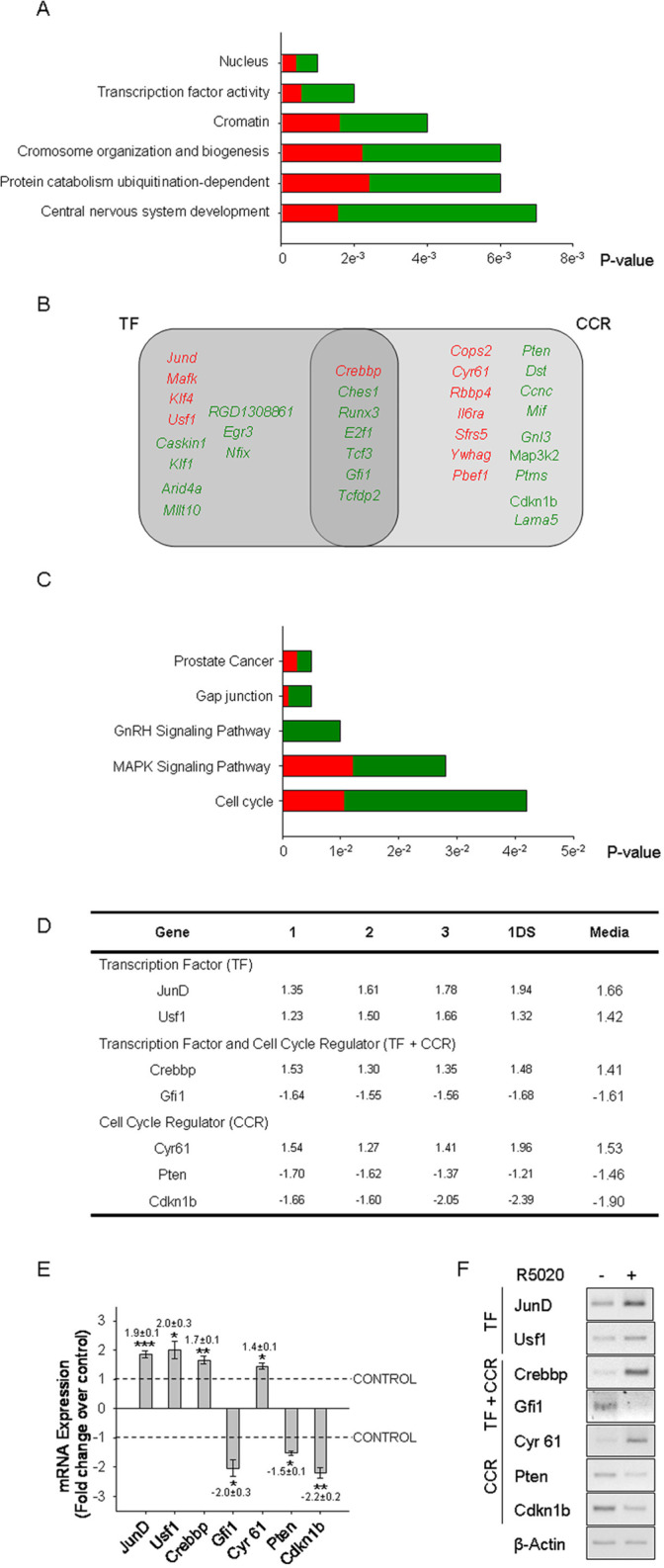
Transcription Factors and Cell Cycle Regulators are the main early progestin-regulated genes. A) The categories of over-represented Gene Ontology terms (GO terms) are shown by their decreasing p-values. The categories were identified by GOTM (Gene Ontology Tree Machine) software over the statistical regulated genes as indicated in supplementary Materials and Methods. Up-regulated genes percentages are shown in red, and down-regulated percentages are shown in green. B) Venn diagram shows the set of TF and CCR regulated by progestin. A GOTM search of cellular proliferation and cell cycle GO terms identified 23 genes, named cell cycle regulators (CCR) in the Figure Seven of them were also categorized as transcription factors (TF) present in A. Up-regulated genes are shown in red, and down-regulated are shown in green. C) The signalling pathways associated to the differential gene expression pattern are shown by their decreasing p-value. Pathways identified by Pathway Express Software containing at least four progestin-dependent regulated genes included in a given Signalling Pathway (SP), with a p-value ≤ 0.05. The percentage of up-regulated genes within a given signalling pathway is shown in red, and down-regulated genes are shown in green. Statistical details are described in M&M. D) The table shows individual fold changes of three independent biological samples (1, 2, 3) and one dye swap data set (1DS) analyzed by microarray, and the mean fold change of all 4 values (Media). Fold changes over vehicle treated cell values were calculated as described in SI M&M. E) q- real time PCR validation for *JunD*, *Usf1*, *Crebbp*, *Gfi1*, *Cyr61*, *Pten* and *Cdkn1b* mRNA relative to β-Actin. The figure shows media ± SEM from three to six independent experiments. *P<0.05, **P<0.01, ***P<0.001 v. vehicle treated cells. F) sq-PCR validation for transcription factors (TF) *JunD*, *Usf1*, transcription factors and cell cycle regulators (TF+CCR) *Crebbp*, *Gfi1*, *Cyr61*, and cell cycle regulators (CCR) *Pten*, *Cdkn1b* and *β-Actin*. Non-adjacent bands from the same gel are indicated with slim solid black lines.

**Fig 4:**
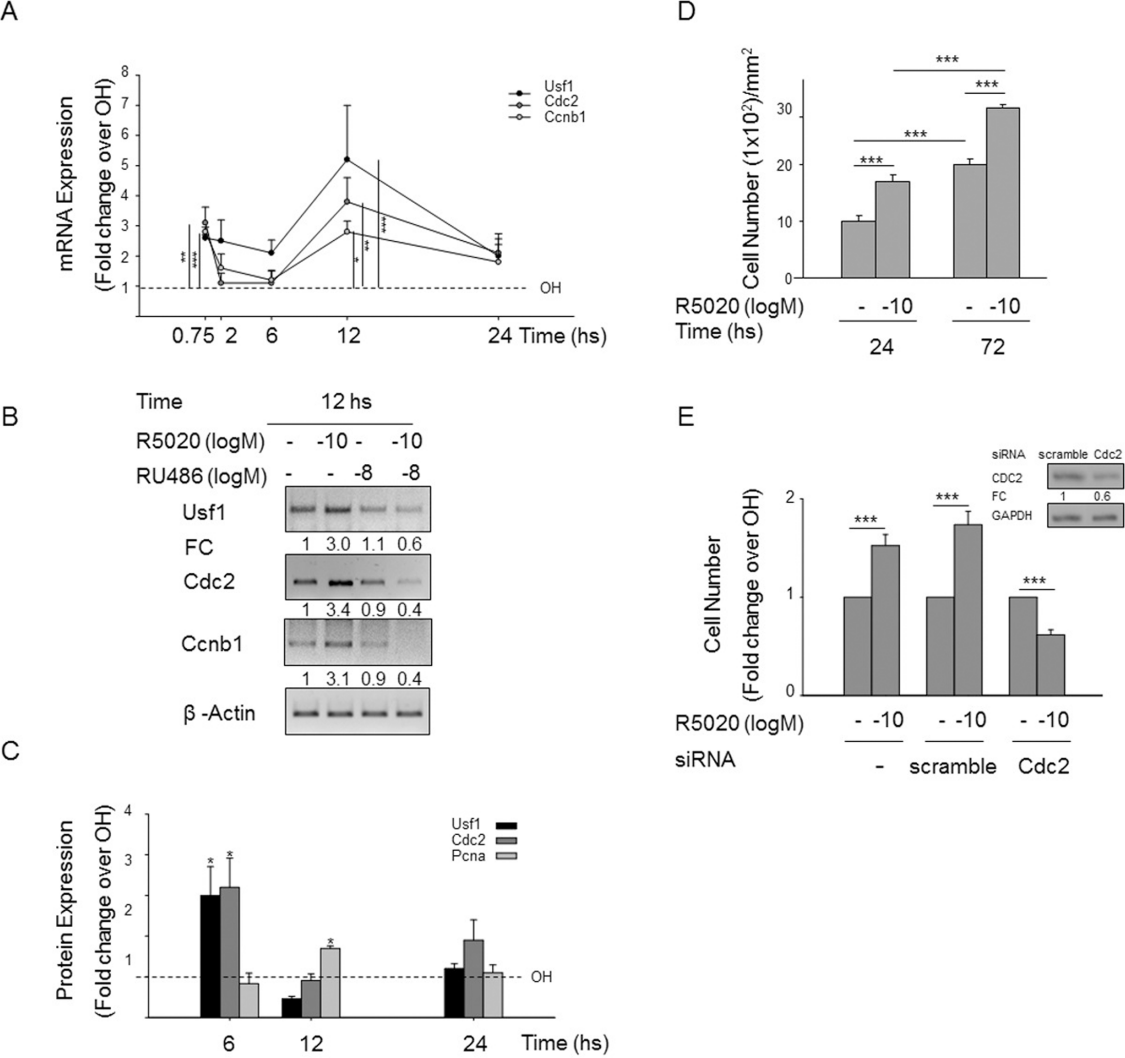
CDC2, a USF1 target, is responsible for progestin-induced UIII cell growth. A) UIII cells were treated at 45 minutes, 2, 6, 12 and 24 h with 10-10 **M** R5020 as indicated in Fig 1A. The values for mRNAs fold change of *Usf1*, *Cdc2*, *Ccnb1* relative to were divided by the vehicle-treated value for each time point tested. Data represent average ± SEM from 3 to 5 independent experiments. * P<0.05, ** P<0.01, *** P<0.001 v. vehicle. **B)** shows representative products of sq-PCR of these genes in 30 minutes RU486 pre-treated cells from three independent experiments with similar results. Non-adjacent bands from the same gel are indicated with slim solid black lines. **C)** shows USF1, CDC2 and PCNA protein expression in which fold change relative to ERK2 was divided by the vehicle-treated value at 6, 12 and 24 h progestin treatment. Data represent average ±SEM from 3 independent experiments. * P<0.05, ** P<0.01, *** P<0.001 v. vehicle. D) Number of cells after 24 and 72 h of vehicle (-) and 10-10M progestin treatments in UIII cells were treated as indicated in Fig 1B. *** P<0.001. E) Number of cells transfected without siRNA, with scramble siRNA and with Cdc2 siRNA 24 h before treatment with vehicle and progestin. *** P<0.001. Lines in both figures indicate statistical comparison; standard deviation is indicated. Inset E) Western blots for CDC2 and GAPDH of cell transfected with Cdc2 and scramble siRNAs.

The available underlying data for Figs 1, 2H and 4B are provided here in S1 and S2 Files. The corresponding author stated that the remainder of the original raw data supporting the figures in the article are available with the exception of the raw data for the Gfi1, Pten and beta Actin gels in Fig 2F.

A member of the *PLOS One* Editorial Board reviewed the underlying data and updated versions of Figs 1, 2 and 4 and stated that they align with the published results and conclusions that progestin regulates endometrial stromal cell proliferation via ERK1–2 and AKT activation independent of PR binding to chromatin.

## Supporting Information

S1 File The available raw image data supporting Figs 1, 2H , and 4B. This includes the underlying Ccnd1 and beta Actin agarose gels from Fig 1A, the underlying Ccnd1 (RU, ICI, PD and LY) and beta Actin (RU, ICI, PD and LY) agarose gels from Fig 1C, the underlying c-Myc, Cdkn1a, beta Actin and Ccnd1 agarose gels from Fig 1D, the underlying JunD, Usf1, Crebbp, Cyr61 and Cdkn1b agarose gels from Fig 2H, and the underlying Cdc2 and Ccnb1agarose gels from Fig 4B.
(ZIP)

S2 File Underlying quantitative data for Fig 1B. (XLS)

## References

[1] Vallejo G, La Greca AD, Tarifa-Reischle IC, Mestre-Citrinovitz AC, Ballaré C, Beato M, et al. CDC2 mediates progestin initiated endometrial stromal cell proliferation: a PR signaling to gene expression independently of its binding to chromatin. PLoS One. 2014;9(5): e97311. doi: 10.1371/journal.pone.0097311 24859236 PMC4032247

