# Supplementary figures and images for: CDC2 Mediates Progestin Initiated Endometrial Stromal Cell Proliferation: A PR Signaling to Gene Expression Independently of Its Binding to Chromatin
Source: PLoS One. 2014 May 23;9(5):e97311. doi: 10.1371/journal.pone.0097311 (PMC4032247; doi:10.1371/journal.pone.0097311)

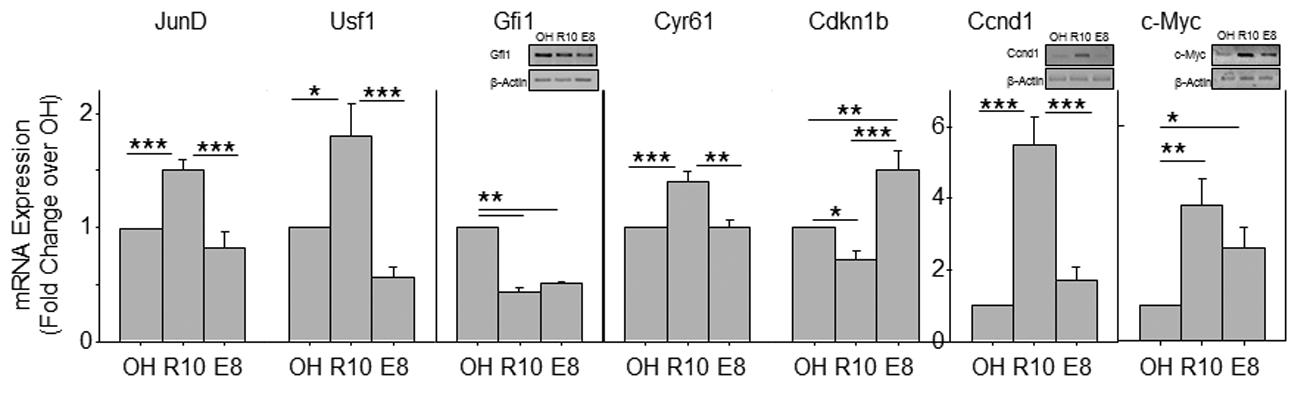

Supplement: Figure S1 — Estradiol effects on validated Transcription Factors and Cell Cycle Regulators. UIII cells were treated as described in Figure 1B and treated with vehicle (OH), R5020 10−10 M (R10) or Estradiol 10−8 M (E8) for 45 minutes in a serum-free culture medium. Graphs show JunD, Usf1, Cyr61, Cdkn1b mRNAs expression determined by q-PCR. Gfi1, Ccnd1 and c-Myc mRNAs expression was analyzed by sq-PCR and representative electrophoresis gels stained with sybr-green are shown in the insets. In all cases values for gene fold change relative to β-Actin were divided by the vehicle-treated value. Data represent average ± SEM from 3–5 independent experiments. *P<0.05, **P<0.01, ***P<0.001. (TIF) [file pone.0097311.s001.tif]

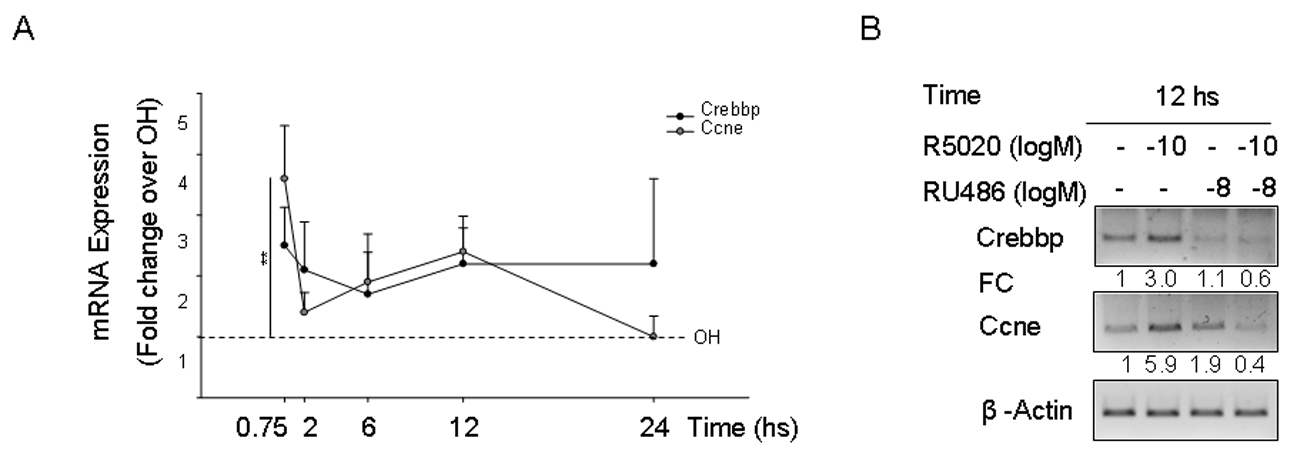

Supplement: Figure S2 — Progestin regulation of Crebbp transcription factor targets. UIII cells were treated as indicated in Fig. 1D. The values for mRNAs fold change relative to β-Actin were divided by the vehicle-treated value for each time point tested. A) Crebbp, CcnE and β-Actin at 45 minutes, 2, 6, 12 and 24 h of 10−10 M R5020. Data represent average ± SEM from 3 to 5 independent experiments. **P<0.01 vs vehicle. B) representative products of sq-PCR of these genes in 30 minutes RU486 pre-treated cells from three independent experiments with similar results. (TIF) [file pone.0097311.s002.tif]
