# Supplementary material for: CDC2 Mediates Progestin Initiated Endometrial Stromal Cell Proliferation: A PR Signaling to Gene Expression Independently of Its Binding to Chromatin
Source: PLoS One. 2014 May 23;9(5):e97311. doi: 10.1371/journal.pone.0097311 (PMC4032247; doi:10.1371/journal.pone.0097311)
Supplement: Table S1 — PCR primer sequences designed by OLIGO Primer Analysis Software (Molecular Biology Insights, Inc.). (DOC) [file pone.0097311.s003.doc]

| **Table S1. PCR Primers** | | | |
| --- | --- | --- | --- |
| Forward |  | Reverse |  |
| **Primer Name** | **Primer Sequence** | **Primer Name** | **Primer Sequence** |
| Ccnd1 | TAGTGTTTAGGTGTGGG | Ccnd1 | GACTGTTCGCTGACTTT |
| c-Myc | ACCTCCAGCCTGTACCTGCA | c-Myc | TGTGTGGAGGTTTGCTGTGG |
| Cdkn1a | CCAGGAGGCCCGAGAACGG | Cdkn1a | CGAGGGGAGGGGCAGGC |
| JunD | GGAAGAGAGAACGGGAGTG | JunD | CGAACCAAGGATTACGGAACA |
| Usf1 | GCTGTCCAAAATCATCCC | Usf1 | GATCTTCCACCTGCTGTC |
| Crebbp | TCTCAATAGTAACTCTGG | Crebbp | GAGAAACCTGTGTCAAAG |
| Gfi | AGGCATCACCGCTTGAAC | Gfi | CCACGGGACCTCTGACTC |
| Cyr61 | CGCGAAGCAACTCAACGAGGA | Cyr61 | GCCCACAGCACCGTCAATACA |
| Pten | AGAGATAAAAAGGGAGTA | Pten | GGCAATGGCTGAGGGAAC |
| Cdkn1b | GGATGAGGAAGCGACC | Cdkn1b | TTCATAAAGCAGTGATGT |
| Cdc2 | CCCGACGAGTTCTTCA | Cdc2 | GCTCCAGATGTCAACC |
| Ccne | TCAACGACACGGGAGAAG | Ccne | GAACCATCCACTTGACAC |
| β-Actin | CCGGGACCTGACAGACTACCT | β-Actin | CCGCTCATTGCCGATAGTGAT |
| Ubs 1 | TTTGGAGCTGAGGACCAAGTG | Ubs 1 | TAAACAAGGCTGGGCTTGAAC |
| nUbs 2 | TTCAAGCCCAGCCTTGTTTA | nUbs 2 | GCTAGCCACAAAAGTGATAATGC |
| Ubs 3 | GACGACATTGGAAGGAAAGC | Ubs 3 | TGCACGTAGACGTTCAAAGG |
| nUbs 4 | CTGGGATCGATCTCCTATGG | nUbs 4 | GGCTCAGAGGAACCATGCTC |
| Ubs 1 bis | ATGCACACACACACACATGC | Ubs 1 bis | CTGAGTGCCCAGGTTAAAGG |

Ubs: USF1 binding site, nUbs: non-USF1 binding site

**Table S1.** PCR primer sequences designed by OLIGO Primer Analysis Software (Molecular Biology Insights, Inc.).
