# Supplementary material for: CDC2 Mediates Progestin Initiated Endometrial Stromal Cell Proliferation: A PR Signaling to Gene Expression Independently of Its Binding to Chromatin
Source: PLoS One. 2014 May 23;9(5):e97311. doi: 10.1371/journal.pone.0097311 (PMC4032247; doi:10.1371/journal.pone.0097311)
Supplement: Table S2 — PCR primers position relative to Cdc2 Transcription Start Site (TSS). Primers Ubs 1 and 3 correspond to region 1 and 3 respectively, while primers nUbs 2 and 4 correspond to regions 2 and 4 respectively in figure 5C. Primers Ubs 1 bis are located just upstream of the Ubs 1 pair and cover a region which partially overlaps with region 1, namely 1 bis. (DOC) [file pone.0097311.s004.doc]

| **Table S2. PCR Primers – relative position to Cdc2 TSS** | | | |
| --- | --- | --- | --- |
| Forward |  | Reverse |  |
| **Primer Name** | **Primer position (bp)** | **Primer Name** | **Primer position (bp)** |
| Ubs 1 | -623 to -603 | Ubs 1 | -472 to -452 |
| nUbs 2 | -471 to -452 | nUbs 2 | -344 to -322 |
| Ubs 3 | -216 to -197 | Ubs 3 | -74 to -55 |
| nUbs 4 | +329 to +348 | nUbs 4 | +463 to +482 |
| Ubs 1 bis | -738 to -719 | Ubs 1 bis | -519 to -500 |

Ubs: USF1 binding site, nUbs: non-USF1 binding site

**Table S2.** PCR primers position relative to Cdc2 Transcription Start Site (TSS). Primers Ubs 1 and 3 correspond to region 1 and 3 respectively, while primers nUbs 2 and 4 correspond to regions 2 and 4 respectively in figure 5C. Primers Ubs 1 bis are located just upstream of the Ubs 1 pair and cover a region which partially overlaps with region 1, namely 1 bis.
