# Supplementary material for: CDC2 Mediates Progestin Initiated Endometrial Stromal Cell Proliferation: A PR Signaling to Gene Expression Independently of Its Binding to Chromatin
Source: PLoS One. 2014 May 23;9(5):e97311. doi: 10.1371/journal.pone.0097311 (PMC4032247; doi:10.1371/journal.pone.0097311)
Supplement: Table S3 — Progestin-dependent up-regulated gene expression pattern. The table shows individual fold changes of up-regulated genes after 45 min treatment with R5020 10−10 M related to vehicle. Data were taken from three independent samples (E1, E2, E3) and one dye swap experiment (1DS) analyzed by microarray and expressed by mean fold change of all 4 values (FC). Colour scale for up (red), non (black) and down (green) regulated genes is shown. (DOC) [file pone.0097311.s005.doc]

**Table S3. Progestin-dependent up-regulated gene expression pattern.**

| **E1** | **E2** | **E3** | **1DS** | **Mean** | **Gene Symbol** | **Gene Name** | **FC** |
| --- | --- | --- | --- | --- | --- | --- | --- |
| # | # | # | # | # | Cirbp | Cold inducible RNA binding protein | **1,95** |
| # | # | # | # | # | AA944679 | Transcribed locus | **1,84** |
| # | # | # | # | # | AA944679 | Transcribed locus | **1,78** |
| # | # | # | # | # | NM_134359 | Protein phosphatase 4 (formerly X), catalytic subunit | **1,71** |
| # | # | # | # | # | Uxs1 | UDP-glucuronate decarboxylase 1 | **1,68** |
| # | # | # | # | # | RGD1311558_predicted | Similar to 4930506M07Rik protein (predicted) | **1,67** |
| # | # | # | # | # | Jund | Jun D proto-oncogene | **1,66** |
| # | # | # | # | # | Ptk9_predicted | Protein tyrosine kinase 9 (predicted) | **1,61** |
| # | # | # | # | # | Entpd5 | Ectonucleoside triphosphate diphosphohydrolase 5 | **1,60** |
| # | # | # | # | # | AA848977 | Transcribed locus | **1,58** |
| # | # | # | # | # | Parg | Poly (ADP-ribose) glycohydrolase | **1,57** |
| # | # | # |  | # | Ppp3r1 | Protein phospatase 3, regulatory subunit B, alpha isoform,type 1 | **1,57** |
| # | # | # | # | # | CB548074 | Necdin-like 2 (predicted) | **1,57** |
| # | # | # | # | # | TC522418 | Unknown | **1,56** |
| # | # | # | # | # | AA998304 | Transcribed locus | **1,56** |
| # | # | # | # | # | NM_001024800 | Thioredoxin domain containing 1 (predicted) | **1,55** |
| # | # | # | # | # | NM_001024310 | Similar to ADP-ribosylation-like factor 6-interacting protein 6 | **1,54** |
| # | # | # | # | # | Cops2 | COP9 (constitutive photomorphogenic) homolog, subunit 2 (Arabidopsis thaliana) | **1,54** |
| # | # | # | # | # | CB545562 | Similar to N-acetylneuraminate pyruvate lyase | **1,54** |
| # | # | # | # | # | Mafk | V-maf musculoaponeurotic fibrosarcoma oncogene family, protein K (avian) | **1,54** |
| # | # | # | # | # | Exosc3_predicted | Exosome component 3 (predicted) | **1,53** |
| # | # | # | # | # | CB546740 | Unknown | **1,53** |
| # | # | # | # | # | Cyr61 | Cysteine rich protein 61 | **1,53** |
| # | # | # | # | # | Rbbp4_predicted | Similar to HECT type E3 ubiquitin ligase | **1,52** |
| # | # | # | # | # | CB557839 | Similar to chr2 synaptotagmin | **1,51** |
| # | # | # | # | # | CA339586 | Potassium channel tetramerisation domain containing 12 (predicted) | **1,51** |
| # | # | # |  | # | Ywhaz | Tyrosine 3-monooxygenase/tryptophan 5-monooxygenase activation protein, zeta polypeptide | **1,51** |
| # | # | # | # | # | TC520968 | Unknown | **1,51** |
| # | # | # |  | # | Rabgef1_predicted | RAB guanine nucleotide exchange factor (GEF) 1 (predicted) | **1,50** |
| # | # | # |  | # | Gdi2 | GDP dissociation inhibitor 2 | **1,50** |
| # | # | # | # | # | Pdcd4 | Programmed cell death 4 | **1,50** |
| # | # | # |  | # | Cyfip1_predicted | Cytoplasmic FMR1 interacting protein 1 (predicted) | **1,49** |
| # | # | # | # | # | Dsipi | Delta sleep inducing peptide, immunoreactor | **1,49** |
| # | # | # | # | # | TC553800 | Unknown | **1,49** |
| # | # | # | # | # | Hspd1 | Hypothetical gene supported by BC086507; NM_022229 | **1,49** |
| # | # | # | # | # | Klf4 | Kruppel-like factor 4 (gut) | **1,49** |
| # | # | # | # | # | Adk | Adenosine kinase | **1,49** |
| # | # | # | # | # | LOC313581 | Similar to CGI-94 protein | **1,49** |
| # | # | # | # | # | Uap1_predicted | Similar to UDP-N-acteylglucosamine pyrophosphorylase 1 homolog | **1,49** |
| # | # | # | # | # | LOC289181 | Similar to IQ motif and WD repeats 1 | **1,49** |
| # | # | # | # | # | NM_001025660 | Etoposide induced 2.4 mRNA (predicted) | **1,49** |
| # | # | # |  | # | Sgpp1 | Sphingosine-1-phosphate phosphatase 1 | **1,48** |
| # | # | # | # | # | Pdgfrb | Hypothetical gene supported by NM_031525 | **1,48** |
| # | # | # | # | # | Tmem33 | Transmembrane protein 33 | **1,48** |
| # | # | # | # | # | Il6r | Interleukin 6 receptor | **1,48** |
| # | # | # |  | # | RGD1307434_predicted | Similar to RIKEN cDNA 2410016F19 (predicted) | **1,48** |
| # | # | # |  | # | Drg1_predicted | Developmentally regulated GTP binding protein 1 (predicted) | **1,47** |
| # | # | # | # | # | Sfrs5 | Splicing factor, arginine/serine-rich 5 | **1,47** |
| # | # | # |  | # | Ptpn12 | Protein tyrosine phosphatase, non-receptor type 12 | **1,47** |
| # | # | # | # | # | TC559256 | Unknown | **1,47** |
| # | # | # |  | # | LOC367184 | Similar to RNA binding motif, single stranded interacting protein 3 | **1,47** |
| # | # | # | # | # | CB545678 | Similar to hypothetical protein 2610304F09 (predicted) | **1,46** |
| # | # | # | # | # | BF557943 | Transcribed locus | **1,46** |
| # | # | # |  | # | XM_576382 | Similar to integral membrane protein 1 | **1,46** |
| # | # | # | # | # | Dnajc9_predicted | DnaJ (Hsp40) homolog, subfamily C, member 9 (predicted) | **1,45** |
| # | # | # | # | # | Snx16 | Sorting nexin 16 | **1,45** |
| # | # | # | # | # | Dnajb9 | DnaJ (Hsp40) homolog, subfamily B, member 9 | **1,45** |
| # | # | # | # | # | Taldo1 | Transaldolase 1 | **1,45** |
| # | # | # | # | # | BF547960 | Transcribed locus | **1,45** |
| # | # | # | # | # | TC537510 | Unknown | **1,44** |
| # | # | # | # | # | AW917546 | Similar to peroxisome proliferator-activated receptor binding protein | **1,44** |
| # | # | # | # | # | AW915598 | Transcribed locus, strongly similar to NP_112577.1 SF3b10 [Homo sapiens] | **1,44** |
| # | # | # |  | # | Tsn | Translin | **1,44** |
| # | # | # | # | # | Ube3a_predicted | Ubiquitin protein ligase E3A (predicted) | **1,44** |
| # | # | # | # | # | XM_574788 | Similar to 5330440M15Rik protein | **1,43** |
| # | # | # |  | # | TC536971 | Unknown | **1,43** |
| # | # | # |  | # | NM_001014111 | Similar to Male-specific lethal 3-like 1 (MSL3-like 1) (Male-specific lethal-3 homolog 1) | **1,43** |
| # | # | # | # | # | AA963979 | UDP-N-acetyl-alpha-D-galactosamine:polypeptide N-acetylgalactosaminyltransferase 1 | **1,43** |
| # | # | # | # | # | Ube2n | Ubiquitin-conjugating enzyme E2N | **1,43** |
| # | # | # | # | # | NM_001014212 | Similar to ribosomal protein L24-like; 60S ribosomal protein L30 isolog; my024 protein; homolog of yeast ribosomal like protein 24 (predicted) | **1,43** |
| # | # | # |  | # | RGD1310427_predicted | Similar to KIAA0090 protein (predicted) | **1,43** |
| # | # | # | # | # | Ywhag | Tyrosine 3-monooxgenase/tryptophan 5-monooxgenase activation protein, gamma polypeptide | **1,43** |
| # | # | # | # | # | XM_215094 | Unknown | **1,42** |
| # | # | # | # | # | NM_001025670 | Similar to hypothetical protein 4933408F15 | **1,42** |
| # | # | # | # | # | BC091439 | Dedicator of cytokinesis 9 | **1,42** |
| # | # | # | # | # | XM_575228 | GA repeat binding protein, beta 1 (predicted) | **1,42** |
| # | # | # | # | # | NM_001024769 | Hyperparathyroidism 2 (with jaw tumor) (predicted) | **1,42** |
| # | # |  | # | # | Arl6ip5 | ADP-ribosylation factor-like 6 interacting protein 5 | **1,42** |
| # | # | # | # | # | RGD:621517 | CD48 antigen | **1,42** |
| # | # | # | # | # | A_42_P711993 | Unknown | **1,42** |
| # | # | # | # | # | Tra1_predicted | Tumor rejection antigen gp96 (predicted) | **1,42** |
| # | # | # | # | # | Usf1 | Upstream transcription factor 1 | **1,42** |
| # | # | # | # | # | Crebbp | Hypothetical gene supported by NM_133381 | **1,41** |
| # | # | # |  | # | Arf2 | ADP-ribosylation factor 2 | **1,41** |
| # | # | # | # | # | Zmynd19 | Zinc finger, MYND domain containing 19 | **1,41** |
| # | # | # |  | # | BE107042 | Transcribed locus | **1,41** |
| # | # | # |  | # | Pdcd8 | Programmed cell death 8 | **1,41** |
| # | # | # | # | # | RGD1311457_predicted | Similar to RIKEN cDNA 2310003F16 (predicted) | **1,41** |
| # | # | # |  | # | Pbef1 | Pre-B-cell colony enhancing factor 1 | **1,41** |
| # | # | # |  | # | NM_001013228 | Similar to T-complex associated-testis-expressed 1-like (Protein 91/23) | **1,41** |
| # | # | # | # | # | LOC294067 | Similar to WW domain binding protein 5 | **1,41** |
| # | # | # | # | # | RGD:1303128 | Zinc finger protein 183 (RING finger, C3HC4 type) | **1,41** |
| # | # | # | # | # | TC521284 | Unknown | **1,40** |
| # | # | # | # | # | AA957447 | Transcribed locus | **1,40** |
| # | # | # | # | # | Cd44 | CD44 antigen | **1,40** |
| # | # | # | # | # | LOC362703 | Similar to WD-repeat protein 43 | **1,40** |
| # | # | # |  | # | Egfl6 | Epidermal growth factor-like protein 6 | **1,40** |


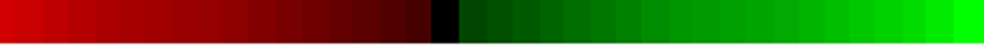


5 1.2 -1.2 -5

Hybridization was performed with samples from 3 independent experiments (E1, E2, E3) and experiment 1 with dye-swap (1DS). FC: mean fold change.

**Table S3. Progestin-dependent up-regulated gene expression pattern.** The table shows individual fold changes of statistical up-regulated genes after 45 min treatment with R5020 10^-10^M related to vehicle. Data were taken from three independent biological samples (E1, E2, E3) and one dye swap sample (1DS) analyzed by microarray and expressed by mean fold change of all 4 values (FC). Up (red) regulated genes are ordered by decreasing FC ≥ 1.4 and a B rank value ≥ 85 (see statistical analysis in M&M). Colour range for 5 ≤FC≥ 1.2 (red scale) and for -1.2 ≥FC≥-5 (green scale) (AFM 4.0) is shown. Black boxes denotes for 1.2 <FC>1.2.
