# Supplementary material for: CDC2 Mediates Progestin Initiated Endometrial Stromal Cell Proliferation: A PR Signaling to Gene Expression Independently of Its Binding to Chromatin
Source: PLoS One. 2014 May 23;9(5):e97311. doi: 10.1371/journal.pone.0097311 (PMC4032247; doi:10.1371/journal.pone.0097311)
Supplement: Table S4 — Progestin-dependent down-regulated gene expression pattern. The table shows individual fold changes of statistical down-regulated genes after 45 min treatment with R5020 10−10 M related to vehicle. Down (green) regulated genes are ordered by increasing mean fold change. Data shown as indicated in Table S3. (DOC) [file pone.0097311.s006.doc]

**Table S4. Progestin-dependent down-regulated gene expression pattern.**

| **E1** | **E2** | **E3** | **1DS** | **Mean** | **Gene Symbol** | **Gene Name** | **FC** |
| --- | --- | --- | --- | --- | --- | --- | --- |
| # | # | # | # | # | RGD1310994_predicted | Similar to polyglutamine-containing protein (predicted) | **-3,89** |
| # | # | # | # | # | TC525207 | Unknown | **-3,26** |
| # | # | # | # | # | RGD:1303284 | Transient receptor potential cation channel subfamily A member 1 | **-2,98** |
| # | # | # | # | # | BE101615 | Unknown | **-2,78** |
| # | # | # | # | # | Adcy7 | Unknown | **-2,74** |
| # | # | # | # | # | Ldhc | Lactate dehydrogenase 3, C chain | **-2,67** |
| # | # | # | # | # | Gcnt2 | Glucosaminyl (N-acetyl) transferase 2, I-branching enzyme | **-2,67** |
| # | # | # | # | # | Atp6v0a4_predicted | ATPase, H+ transporting, lysosomal V0 subunit A isoform 4 (predicted) | **-2,63** |
| # | # | # | # | # | A_43_P14037 | Unknown | **-2,62** |
| # | # | # | # | # | Srpr_predicted | Signal recognition particle receptor ('docking protein') (predicted) | **-2,56** |
| # | # | # | # | # | TC552253 | Unknown | **-2,51** |
| # | # | # | # | # | Pitpnm_predicted | Phosphatidylinositol membrane-associated (predicted) | **-2,51** |
| # | # | # | # | # | Polm_predicted | Polymerase (DNA directed), mu (predicted) | **-2,48** |
| # | # | # | # | # | RGD1306107_predicted | Similar to chromosome 1 open reading frame 2 (predicted) | **-2,43** |
| # | # | # | # | # | TC549240 | Unknown | **-2,33** |
| # | # | # | # | # | NM_001024790 | Ubiquitin specific protease 7 (herpes virus-associated) (predicted) | **-2,30** |
| # | # | # | # | # | CA338760 | Unknown | **-2,25** |
| # | # | # | # | # | A_43_P13853 | Unknown | **-2,22** |
| # | # | # | # | # | Fxyd7 | FXYD domain-containing ion transport regulator 7 | **-2,20** |
| # | # | # | # | # | A_42_P824877 | Unknown | **-2,20** |
| # | # | # | # | # | TC560016 | Unknown | **-2,16** |
| # | # | # | # | # | A_43_P22350 | Unknown | **-2,15** |
| # | # | # | # | # | Lama5 | Laminin, alpha 5 | **-2,12** |
| # | # | # | # | # | LOC288978 | Similar to RIKEN cDNA 3110009E18 | **-2,11** |
| # | # | # | # | # | Pnutl2_predicted | Peanut-like 2 (Drosophila) (predicted) | **-2,10** |
| # | # | # | # | # | A_43_P19353 | Unknown | **-2,10** |
| # | # | # | # | # | XM_344782 | PREDICTED: Rattus norvegicus zinc finger protein 469 (predicted) (Znf469_predicted), mRNA | **-2,09** |
| # | # | # | # | # | BF562492 | Rho-related BTB domain containing 3 (predicted) | **-2,09** |
| # | # | # | # | # | RGD1311929_predicted | Similar to hypothetical protein D11Ertd707e (predicted) | **-2,08** |
| # | # | # | # | # | LOC290876 | Similar to RIKEN cDNA 1700029H14 | **-2,07** |
| # | # | # | # | # | A_43_P14272 | Unknown | **-2,07** |
| # | # | # | # | # | LOC360760 | Similar to OTTHUMP00000042400 | **-2,06** |
| # | # | # | # | # | LOC289617 | Similar to chromaffin granule ATPase II homolog | **-2,05** |
| # | # | # | # | # | Luzp1 | Leucine zipper protein 1 | **-2,03** |
| # | # | # | # | # | Abcc10_predicted | ATP-binding cassette, sub-family C (CFTR/MRP), member 10 (predicted) | **-2,01** |
| # | # | # | # | # | BF562776 | Transcribed locus | **-2,01** |
| # | # | # | # | # | Kif9_predicted | Kinesin family member 9 (predicted) | **-1,99** |
| # | # | # | # | # | Enh | Enigma homolog | **-1,99** |
| # | # | # | # | # | LOC362736 | Similar to HECT domain containing 1 | **-1,98** |
| # | # | # | # | # | BQ783112 | Protein tyrosine phosphatase, non-receptor type 18 (predicted) | **-1,96** |
| # | # | # | # | # | TC555141 | Unknown | **-1,96** |
| # | # | # | # | # | CB545321 | Transcribed locus | **-1,95** |
| # | # | # | # | # | A_42_P585716 | Unknown | **-1,94** |
| # | # | # | # | # | A_43_P11352 | Unknown | **-1,93** |
| # | # | # | # | # | A_43_P22616 | Unknown | **-1,91** |
| # | # | # | # | # | Cdkn1b | Cyclin-dependent kinase inhibitor 1B | **-1,90** |
| # | # | # | # | # | LOC360509 | Similar to HCH | **-1,89** |
| # | # | # | # | # | RGD:1303289 | SCIRP10-related protein | **-1,87** |
| # | # | # | # | # | A_42_P505153 | Unknown | **-1,87** |
| # | # | # | # | # | A_43_P10268 | Unknown | **-1,87** |
| # | # | # | # | # | ENSRNOT00000002071 | Unknown | **-1,86** |
| # | # | # | # | # | X67303 | POU domain, class 2, transcription factor 3 | **-1,85** |
| # | # | # | # | # | Mllt10_predicted | LOC498801 | **-1,85** |
| # | # | # | # | # | CB605764 | General transcription factor IIIC, polypeptide 5 (predicted) | **-1,85** |
| # | # | # | # | # | TC524581 | Unknown | **-1,83** |
| # | # | # | # | # | CB544837 | Son of sevenless homolog 1 (Drosophila) (predicted) | **-1,83** |
| # | # | # | # | # | Ep400_predicted | E1A binding protein p400 (predicted) | **-1,83** |
| # | # | # | # | # | XM_342519 | Unknown | **-1,82** |
| # | # | # | # | # | Ptms | Parathymosin | **-1,81** |
| # | # | # | # | # | TC552159 | Unknown | **-1,81** |
| # | # | # | # | # | Gnl3 | Guanine nucleotide binding protein-like 3 (nucleolar) | **-1,81** |
| # | # | # | # | # | Map3k2 | Unknown | **-1,81** |
| # | # | # | # | # | NM_001014007 | Hypothetical LOC306766 | **-1,80** |
| # | # | # | # | # | BF563566 | Unknown (protein for MGC:72974) | **-1,79** |
| # | # | # | # | # | RGD1306245_predicted | Similar to CLIP-170-related protein (predicted) | **-1,78** |
| # | # | # | # | # | TC550581 | Unknown | **-1,78** |
| # | # | # | # | # | Spnb4_predicted | Spectrin beta 4 (predicted) | **-1,78** |
| # | # | # | # | # | LOC309692 | Similar to Disco-interacting protein 2 homolog | **-1,77** |
|  | # | # |  | # | A_43_P23442 | Unknown | **-1,77** |
| # | # | # | # | # | TC557127 | Unknown | **-1,77** |
| # | # | # | # | # | LOC306622 | Hypothetical LOC306622 | **-1,77** |
| # | # | # | # | # | Tpr_predicted | Translocated promoter region (predicted) | **-1,76** |
| # | # | # | # | # | RGD1311624_predicted | Similar to KIAA0339 protein (predicted) | **-1,75** |
| # | # | # | # | # | AW143673 | Tweety homolog 3 (Drosophila) (predicted) | **-1,74** |
| # | # | # | # | # | M15650 | Unknown | **-1,74** |
| # | # | # | # | # | CB545888 | Unknown | **-1,74** |
| # | # | # | # | # | TC540933 | Unknown | **-1,73** |
| # | # | # | # | # | S100a8 | S100 calcium binding protein A8 (calgranulin A) | **-1,72** |
| # | # | # | # | # | Ptprg | Protein tyrosine phosphatase, receptor type, G | **-1,72** |
| # | # | # | # | # | A_43_P19951 | Unknown | **-1,72** |
| # | # | # | # | # | RGD1304626_predicted | Similar to KIAA1128 protein (predicted) | **-1,72** |
| # | # | # | # | # | NM_001013167 | WAS protein family, member 2 (predicted) | **-1,71** |
| # | # | # | # | # | Dnajc5 | Cysteine string protein | **-1,70** |
| # | # | # | # | # | TC539990 | Unknown | **-1,70** |
| # | # | # | # | # | Sst | Somatostatin | **-1,70** |
| # | # | # | # | # | Brd1_predicted | Bromodomain containing 1 (predicted) | **-1,70** |
| # | # | # | # | # | LOC307395 | Similar to mKIAA0843 protein | **-1,70** |
| # | # | # | # | # | CB546393 | Similar to CG17660-PA (predicted) | **-1,69** |
| # | # | # | # | # | Rps10 | Ribosomal protein S10 | **-1,69** |
| # | # | # | # | # | Ifnar1_predicted | Interferon (alpha and beta) receptor 1 (predicted) | **-1,69** |
| # | # | # | # | # | CB547060 | Similar to hypothetical protein | **-1,68** |
| # | # | # | # | # | CB606030 | Similar to 2010004A03Rik protein | **-1,68** |
| # | # | # | # | # | Edg2 | Endothelial differentiation, lysophosphatidic acid G-protein-coupled receptor, 2 | **-1,68** |
| # | # | # | # | # | Pmfbp1 | Polyamine modulated factor 1 binding protein 1 | **-1,68** |
| # | # | # | # | # | CB546435 | CDNA clone MGC:93962 IMAGE:7114273 | **-1,68** |
| # | # | # | # | # | A_42_P524225 | Unknown | **-1,68** |
| # | # | # | # | # | BF562817 | Unknown | **-1,68** |
| # | # | # | # | # | Zcchc11_predicted | Zinc finger, CCHC domain containing 11 (predicted) | **-1,67** |
| # | # | # | # | # | TC521012 | Unknown | **-1,67** |
| # | # | # | # | # | CB546979 | Unknown | **-1,67** |
| # | # | # | # | # | Grca_predicted | Gene rich cluster, A gene (predicted) | **-1,66** |
| # | # | # | # | # | CB547875 | Dentatorubral pallidoluysian atrophy | **-1,66** |
| # | # | # | # | # | TC535019 | Unknown | **-1,66** |
| # | # | # | # | # | TC555422 | Unknown | **-1,66** |
| # | # | # | # | # | Colm | Collomin | **-1,66** |
| # | # | # | # | # | TC556715 | Unknown | **-1,65** |
| # | # | # | # | # | A_42_P790250 | Unknown | **-1,65** |
| # | # | # | # | # | Gprin1_predicted | G protein-regulated inducer of neurite outgrowth 1 (predicted) | **-1,64** |
| # | # | # | # | # | RGD1307882_predicted | Similar to CG9346-PA (predicted) | **-1,64** |
| # | # | # | # | # | Avp | Arginine vasopressin | **-1,64** |
| # | # | # | # | # | AW143544 | Retinol dehydrogenase 10 (all-trans) | **-1,64** |
| # | # | # | # | # | RGD1311486_predicted | Similar to RIKEN cDNA C630028L02 gene (predicted) | **-1,64** |
| # | # | # | # | # | Arid4a_predicted | AT rich interactive domain 4A (Rbp1 like) (predicted) | **-1,63** |
| # | # | # | # | # | A_43_P13718 | Unknown | **-1,63** |
| # | # | # | # | # | Cacng2 | Calcium channel, voltage-dependent, gamma subunit 2 | **-1,63** |
| # | # | # | # | # | RGD:1303152 | Ectodermal-neural cortex 1 | **-1,63** |
| # | # | # | # | # | TC523913 | Unknown | **-1,63** |
| # | # | # | # | # | Pole | Polymerase (DNA directed), epsilon | **-1,63** |
| # | # | # | # | # | A_42_P826907 | Unknown | **-1,62** |
| # | # | # | # | # | Slc2a6_predicted | Solute carrier family 2 (facilitated glucose transporter), member 6 (predicted) | **-1,62** |
| # | # | # | # | # | RGD1311502_predicted | Similar to stonin 2; homolog of stoned B (Drosophila) (predicted) | **-1,62** |
| # | # | # | # | # | CB545634 | Tau tubulin kinase 1 (predicted) | **-1,62** |
| # | # | # | # | # | TC541365 | Unknown | **-1,62** |
| # | # | # | # | # | LOC363135 | Similar to steroid receptor-interacting SNF2 domain protein | **-1,62** |
| # | # | # | # | # | S80379 | Unknown | **-1,62** |
| # | # | # | # | # | Tfdp2_predicted | Transcription factor Dp-2 (E2F dimerization partner 2) (predicted) | **-1,61** |
| # | # | # | # | # | A_43_P15725 | Unknown | **-1,61** |
| # | # | # | # | # | CB547414 | Ciliary rootlet coiled-coil, rootletin (predicted) | **-1,61** |
| # | # |  | # | # | Prpf8_predicted | Pre-mRNA processing factor 8 (predicted) | **-1,61** |
| # | # | # | # | # | LOC315595 | Similar to Poliovirus receptor-related 1 | **-1,61** |
| # | # | # | # | # | Dhx29_predicted | DEAH (Asp-Glu-Ala-His) box polypeptide 29 (predicted) | **-1,61** |
| # | # | # | # | # | Gfi1 | Growth factor independent 1 | **-1,61** |
| # | # | # | # | # | NM_001014154 | Similar to RIKEN cDNA 4930457P18 (predicted) | **-1,61** |
| # | # | # | # | # | Azi1_predicted | 5-azacytidine induced gene 1 (predicted) | **-1,60** |
| # | # | # | # | # | MGC95311 | Similar to RIKEN cDNA 2310005B10 | **-1,60** |
| # | # | # | # | # | Usp25_predicted | Ubiquitin specific protease 25 (predicted) | **-1,60** |
| # | # | # | # | # | Dhx36_predicted | DEAH (Asp-Glu-Ala-His) box polypeptide 36 (predicted) | **-1,60** |
| # | # | # | # | # | Cast | Calpastatin | **-1,59** |
| # | # | # | # | # | TC519851 | Unknown | **-1,59** |
| # | # | # | # | # | TC557022 | Unknown | **-1,59** |
| # | # | # | # | # | Mif | Macrophage migration inhibitory factor | **-1,59** |
| # | # | # | # | # | AABR03024169 | Unknown | **-1,58** |
| # | # | # | # | # | Klf1_predicted | Kruppel-like factor 1 (erythroid) (predicted) | **-1,58** |
| # | # | # | # | # | TC527133 | Unknown | **-1,58** |
| # | # | # | # | # | A_43_P14269 | Unknown | **-1,57** |
| # | # | # | # | # | Sf4_predicted | Splicing factor 4 (predicted) | **-1,57** |
| # | # | # | # | # | BF387255 | Similar to T-cell activation protein phosphatase 2C (predicted) | **-1,56** |
| # | # | # | # | # | AW917070 | Hypothetical LOC304650 (predicted) | **-1,56** |
| # | # | # | # | # | RGD1305162_predicted | Similar to hypothetical protein BC008163 (predicted) | **-1,56** |
| # | # | # | # | # | LOC363517 | Similar to Plexin B3 precursor (Plexin 6) | **-1,56** |
| # | # | # | # | # | TC539638 | Unknown | **-1,56** |
| # | # | # | # | # | Eif3s10_predicted | Eukaryotic translation initiation factor 3, subunit 10 (theta) (predicted) | **-1,56** |
| # | # | # | # | # | Cbl27 | Androgen receptor-related apoptosis-associated protein CBL27 | **-1,56** |
| # | # | # | # | # | LOC317178 | Similar to ubiquitously transcribed tetratricopeptide repeat gene, X chromosome | **-1,56** |
| # | # | # | # | # | Ccnc |  | **-1,56** |
| # | # | # | # | # | Caskin1 | Cask-interacting protein 1 | **-1,56** |
| # | # | # | # | # | TC524942 |  | **-1,55** |
| # | # | # | # | # | NM_001025275 | Similar to MHR23B | **-1,55** |
| # | # | # |  | # | Znf292 | Zinc finger protein 292 | **-1,55** |
| # | # | # | # | # | Zcchc11_predicted | Zinc finger, CCHC domain containing 11 (predicted) | **-1,55** |
| # | # | # | # | # | Ecm1 | Extracellular matrix protein 1 | **-1,55** |
| # | # | # | # | # | Plxnb1_predicted | Plexin B1 (predicted) | **-1,55** |
| # | # | # | # | # | Usp42_predicted | Ubiquitin specific protease 42 (predicted) | **-1,55** |
| # | # | # | # | # | CB605593 | GLI-Kruppel family member GLI3 | **-1,55** |
| # | # | # | # | # | A_42_P727103 |  | **-1,55** |
| # | # | # | # | # | Gstm3 | Glutathione S-transferase, mu type 3 | **-1,55** |
| # | # | # | # | # | TC525104 |  | **-1,54** |
| # | # | # | # | # | Pkp4_predicted | Plakophilin 4 (predicted) | **-1,54** |
| # | # | # | # | # | LOC361899 | Similar to RIKEN cDNA 2610528A15 | **-1,54** |
| # | # | # | # | # | AABR03063179 |  | **-1,54** |
| # | # | # | # | # | BF566330 | Similar to ribosomal protein L21 | **-1,54** |
| # | # | # | # | # | XM_578842 | Similar to RIKEN cDNA 4921515A04 | **-1,54** |
| # | # | # | # | # | AI169579 |  | **-1,54** |
| # | # | # | # | # | TC523436 |  | **-1,54** |
| # | # | # | # | # | Mmp14 | Matrix metalloproteinase 14 (membrane-inserted) | **-1,54** |
| # | # | # | # | # | AA956317 | Synaptogenesis-related mRNA sequence 2, 3' untranslated region | **-1,54** |
| # | # | # | # | # | Tcf3_predicted | Transcription factor 3 (predicted) | **-1,54** |
| # | # | # | # | # | TC534614 |  | **-1,53** |
| # | # | # | # | # | TC540754 |  | **-1,53** |
| # | # | # | # | # | A_43_P22511 |  | **-1,53** |
| # | # | # | # | # | Mras | Muscle and microspikes RAS | **-1,53** |
| # | # | # | # | # | CB545525 | Hypothetical gene supported by NM_181475 | **-1,53** |
| # | # | # | # | # | LOC360865 | Similar to KIAA1096 protein | **-1,53** |
| # | # | # | # | # | Gpr88 | G-protein coupled receptor 88 | **-1,53** |
| # | # | # | # | # | CB547926 | Similar to mKIAA0190 protein | **-1,53** |
| # | # | # | # | # | Abcc1 | Similar to ATP-binding cassette, sub-family C (CFTR/MRP), member 1 | **-1,53** |
| # | # | # | # | # | Enpp1 | Ectonucleotide pyrophosphatase/phosphodiesterase 1 | **-1,52** |
| # | # | # |  | # | LOC311362 | Similar to MGC14161 protein | **-1,52** |
| # | # | # | # | # | XM_228865 |  | **-1,52** |
| # | # | # | # | # | LOC360999 | Similar to RIKEN cDNA 5730466H23 | **-1,52** |
| # | # | # | # | # | TC519315 |  | **-1,52** |
| # | # | # | # | # | CB546606 | Transcribed locus, strongly similar to XP_215761.3 PREDICTED: similar to Actin, cytoplasmic 2 (Gamma-actin) [Rattus norvegicus] | **-1,52** |
| # | # | # | # | # | Lars_predicted | Leucyl-tRNA synthetase (predicted) | **-1,52** |
| # | # | # | # | # | XM_341133 | Laminin, gamma 1 | **-1,52** |
| # | # | # | # | # | Cnot3_predicted | CCR4-NOT transcription complex, subunit 3 (predicted) | **-1,51** |
| # | # | # | # | # | CB545557 | Speckle-type POZ protein (predicted) | **-1,51** |
| # | # | # | # | # | NM_001025735 | Transcription elongation factor A (SII) 1 (predicted) | **-1,51** |
| # | # | # | # | # | Arih2_predicted | Ariadne homolog 2 (Drosophila) (predicted) | **-1,51** |
| # | # |  | # | # | BF548232 | ELAV (embryonic lethal, abnormal vision, Drosophila)-like 1 (Hu antigen R) (predicted) | **-1,51** |
| # | # | # | # | # | BF414362 | Transcribed locus, weakly similar to NP_001002778.1 hypothetical protein LOC442814 [Mus musculus] | **-1,51** |
| # | # | # | # | # | RGD:1303025 | HLA-B associated transcript 2 | **-1,51** |
| # | # | # | # | # | A_43_P14253 |  | **-1,51** |
| # | # |  | # | # | TC544588 |  | **-1,51** |
| # | # | # | # | # | TC523266 |  | **-1,51** |
| # | # | # | # | # | Mmp14 | Matrix metalloproteinase 14 (membrane-inserted) | **-1,50** |
| # | # | # | # | # | Klrc2 | Killer cell lectin-like receptor subfamily C, member 2 | **-1,50** |
| # | # | # | # | # | A_43_P10943 |  | **-1,50** |
| # | # | # | # | # | Dsp_predicted | Desmoplakin (predicted) | **-1,50** |
| # | # | # | # | # | Kcc4 | K-Cl cotransporter KCC4 | **-1,50** |
| # | # | # | # | # | Dst_predicted | Dystonin (predicted) | **-1,50** |
| # | # | # | # | # | Tpr_predicted | Translocated promoter region (predicted) | **-1,50** |
| # | # | # | # | # | RGD1309643_predicted | Similar to RIKEN cDNA C330006K01 (predicted) | **-1,50** |
| # | # | # | # | # | Atrx | Alpha thalassemia/mental retardation syndrome X-linked homolog (human) | **-1,50** |
| # | # | # | # | # | CB547659 |  | **-1,50** |
| # | # | # | # | # | A_43_P14886 |  | **-1,49** |
| # | # | # | # | # | TC526576 |  | **-1,49** |
| # | # | # | # | # | E2f1_predicted | E2F transcription factor 1 | **-1,49** |
| # | # | # | # | # | Runx3 | Runt-related transcription factor 3 | **-1,49** |
| # | # | # |  | # | XM_223060 | Centromere autoantigen F | **-1,49** |
| # | # | # | # | # | AW914967 | Transcribed locus | **-1,48** |
| # | # | # |  | # | Rb1cc1_predicted | RB1-inducible coiled-coil 1 (predicted) | **-1,48** |
| # | # | # | # | # | Ches1_predicted | Checkpoint suppressor 1 (predicted) | **-1,48** |
| # | # | # | # | # | XM_576570 | Similar to KIAA1034-like DNA binding protein | **-1,48** |
| # | # | # |  | # | Setdb1_predicted | SET domain, bifurcated 1 (predicted) | **-1,48** |
| # | # | # | # | # | TC524979 |  | **-1,48** |
| # | # | # | # | # | A_43_P17206 |  | **-1,48** |
| # | # | # | # | # | Tnks1bp1_predicted | Tankyrase 1 binding protein 1 (predicted) | **-1,48** |
| # | # | # | # | # | RGD1309692_predicted | Similar to CG12054-PA (predicted) | **-1,48** |
| # | # | # | # | # | Phf3_predicted | PHD finger protein 3 (predicted) | **-1,48** |
| # | # | # | # | # | Hspb9_predicted | Heat shock protein, alpha-crystallin-related, B9 (predicted) | **-1,48** |
| # | # | # | # | # | TC537775 |  | **-1,48** |
| # | # | # | # | # | RGD1307284_predicted | Similar to protein kinase, lysine deficient 1; kinase deficient protein (predicted) | **-1,48** |
| # | # | # |  | # | H1f4 | H1 histone family, member 4 | **-1,47** |
| # | # | # | # | # | RGD:735140 | Hypothetical protein LK44 | **-1,47** |
| # | # | # | # | # | CA506887 | MYST histone acetyltransferase (monocytic leukemia) 3 (predicted) | **-1,47** |
| # | # | # | # | # | CB545642 |  | **-1,47** |
| # | # | # | # | # | Gys1_predicted |  | **-1,47** |
| # | # | # | # | # | TC537100 |  | **-1,47** |
| # | # | # | # | # | Ate1_predicted | Arginine-tRNA-protein transferase 1 (predicted) | **-1,47** |
| # | # | # | # | # | CB548448 | Zinc finger protein 289 (predicted) | **-1,47** |
| # | # | # | # | # | Herc1_predicted | Hect (homologous to the E6-AP (UBE3A) carboxyl terminus) domain and RCC1 (CHC1)-like domain (RLD) 1 (predicted) | **-1,46** |
| # | # | # | # | # | TC543339 |  | **-1,46** |
| # | # | # | # | # | TC540896 |  | **-1,46** |
| # | # | # | # | # | Pten | Phosphatase and tensin homolog | **-1,46** |
| # | # | # | # | # | Zfyve20_predicted | Zinc finger, FYVE domain containing 20 (predicted) | **-1,46** |
| # | # | # | # | # | A_43_P18808 |  | **-1,46** |
| # | # | # | # | # | Nfix | Nuclear factor I/X | **-1,46** |
| # | # | # | # | # | Egr3 | Early growth response 3 | **-1,46** |
| # | # | # |  | # | BF552424 | Transcribed locus, moderately similar to NP_080479.1 jouberin [Mus musculus] | **-1,46** |
| # | # | # | # | # | AI136550 | PCTAIRE-motif protein kinase 2 | **-1,46** |
| # | # | # | # | # | Pitx3 | Paired-like homeodomain transcription factor 3 | **-1,46** |
|  |  | # |  | # | Rag1_predicted | Recombination activating gene 1 | **-1,46** |
| # | # | # | # | # | Tyrobp | Tyro protein tyrosine kinase binding protein | **-1,46** |
| # | # | # |  | # | XM_574695 | Similar to oocyte-testis gene 1 | **-1,46** |
| # | # | # | # | # | Trim9 | Tripartite motif-containing 9 | **-1,46** |
| # | # | # | # | # | A_42_P468371 |  | **-1,45** |
| # | # | # | # | # | RGD1311993_predicted | Hypothetical LOC315072 (predicted) | **-1,45** |
| # | # | # |  | # | Zbtb8_predicted | Zinc finger and BTB domain containing 8 (predicted) | **-1,45** |
| # | # | # | # | # | Gbe1_predicted | Glucan (1,4-alpha-), branching enzyme 1 (predicted) | **-1,45** |
| # | # | # | # | # | Cst8 | Cystatin 8 | **-1,45** |
| # | # | # | # | # | Smarcc1_predicted | SWI/SNF related, matrix associated, actin dependent regulator of chromatin, subfamily c, member 1 (predicted) | **-1,45** |
| # | # | # | # | # | Smpx | Small muscle protein, X-linked | **-1,45** |
| # | # | # | # | # | Odz2 | Hypothetical gene supported by NM_020088 | **-1,45** |
| # | # | # | # | # | eplin | Epithelial protein lost in neoplasm | **-1,44** |
| # | # |  | # | # | AA946486 | Transcribed locus | **-1,44** |
| # | # | # | # | # | TC558157 |  | **-1,44** |
| # | # | # |  | # | XM_577054 | Similar to 2610030H06Rik protein | **-1,44** |
| # | # | # | # | # | Folr1 | Folate receptor 1 (adult) | **-1,44** |
| # | # |  | # | # | Ddx27_predicted | DEAD (Asp-Glu-Ala-Asp) box polypeptide 27 (predicted) | **-1,44** |
| # | # | # | # | # | TC538981 |  | **-1,44** |
| # | # | # | # | # | LOC363009 | Similar to mKIAA0701 protein | **-1,44** |
| # | # | # | # | # | Rpl39 | Insulin-like growth factor binding protein 7 (predicted) | **-1,44** |
| # | # | # | # | # | RGD1311084_predicted | Similar to 1700113K14Rik protein (predicted) | **-1,44** |
| # | # | # | # | # | A_43_P22440 |  | **-1,44** |
| # | # | # |  | # | RICS_predicted | RhoGAP involved in beta-catenin-N-cadherin and NMDA receptor signaling (predicted) | **-1,44** |
| # | # | # | # | # | CB548473 | Fas-activated serine/threonine kinase (predicted) | **-1,44** |
| # | # | # | # | # | NM_001014207 | Similar to RIKEN cDNA 4930553M18 (predicted) | **-1,44** |
| # | # | # | # | # | RGD1308861_predicted | Similar to protein phosphatase 1, regulatory subunit 12C; myosin-binding subunit 85 (predicted) | **-1,44** |
| # | # | # | # | # | RGD1305857_predicted | Similar to hypothetical protein FLJ13511 (predicted) | **-1,44** |
| # | # | # | # | # | Slc25a29_predicted | Solute carrier family 25 (mitochondrial carrier, palmitoylcarnitine transporter), member 29 (predicted) | **-1,43** |
| # | # | # | # | # | LOC315732 | Similar to Nuclear membrane binding protein NUCLING | **-1,43** |
| # | # | # | # | # | MGC94720 | Similar to hypothetical protein MGC2574 | **-1,43** |
| # | # | # | # | # | A_43_P16839 |  | **-1,43** |
| # | # | # | # | # | MGC94736 | Similar to hypothetical protein MGC35097 | **-1,43** |
| # | # | # | # | # | A_43_P23100 |  | **-1,43** |
| # | # | # | # | # | Gpr6 | G protein-coupled receptor 6 | **-1,43** |
| # | # | # | # | # | TC542996 |  | **-1,43** |
| # | # | # | # | # | U75924 |  | **-1,43** |
| # | # | # | # | # | XM_573559 | BMP-2 inducible kinase | **-1,43** |
| # | # | # | # | # | B3gnt5 | UDP-GlcNAc:betaGal beta-1,3-N-acetylglucosaminyltransferase 5 | **-1,43** |
| # | # | # | # | # | TC559048 |  | **-1,43** |
| # | # | # | # | # | Apoa5 | Apolipoprotein A-V | **-1,43** |
| # | # | # | # | # | TC542847 |  | **-1,42** |
| # | # | # | # | # | TC546666 |  | **-1,42** |
| # | # | # | # | # | Sacs_predicted | Sacsin (predicted) | **-1,42** |
| # | # | # | # | # | Dnah7 | Dynein, axonemal, heavy polypeptide 7 | **-1,42** |
| # | # | # | # | # | Prx | Hypothetical gene supported by NM_023976 | **-1,42** |
| # | # |  | # | # | Lyz | Lysozyme | **-1,42** |
| # | # | # | # | # | LOC361373 | Similar to cylindromatosis (turban tumor syndrome) | **-1,42** |
| # | # | # |  | # | LOC361605 | Similar to mKIAA0824 protein | **-1,42** |
| # | # | # | # | # | TC534856 |  | **-1,42** |
| # | # | # | # | # | Extl1_predicted | Exostoses (multiple)-like 1 (predicted) | **-1,42** |
| # | # | # | # | # | CB546804 | CDNA clone IMAGE:7366251 | **-1,42** |
| # | # | # | # | # | AW144651 |  | **-1,42** |
| # | # | # | # | # | A_42_P817185 |  | **-1,42** |
| # | # | # |  | # | XM_573625 |  | **-1,42** |
| # | # | # | # | # | AW916109 | LOC498010 | **-1,42** |
| # | # | # | # | # | CB548390 |  | **-1,42** |
| # | # | # | # | # | RGD1305204_predicted | Similar to hypothetical protein FLJ20315 (predicted) | **-1,42** |
| # | # | # | # | # | Cdh8 | Cadherin 8 | **-1,41** |
| # | # | # | # | # | A_43_P17637 |  | **-1,41** |
| # | # | # | # | # | Akap9 | A kinase (PRKA) anchor protein (yotiao) 9 | **-1,41** |
| # | # | # | # | # | TC559584 |  | **-1,41** |
| # | # | # | # | # | A_43_P22671 |  | **-1,41** |
| # | # | # | # | # | Epb4.1l4b_predicted | Similar to EHM2 | **-1,41** |
| # | # | # | # | # | Chn2 | Chimerin (chimaerin) 2 | **-1,41** |
| # | # | # | # | # | A1bg | Alpha-1-B glycoprotein | **-1,41** |
| # | # | # | # | # | LOC310463 | Similar to Hypothetical protein MGC57096 | **-1,41** |
| # | # | # | # | # | Zfr_predicted | Zinc finger RNA binding protein (predicted) | **-1,41** |
| # | # | # | # | # | Arhgef1 | Rho guanine nucleotide exchange factor (GEF) 1 | **-1,41** |
| # | # | # | # | # | TC540805 |  | **-1,41** |
| # | # | # | # | # | Plekhc1_predicted | Pleckstrin homology domain containing, family C (with FERM domain) member 1 (predicted) | **-1,40** |
| # | # | # | # | # | ENSRNOT00000050774 |  | **-1,40** |
| # | # | # | # | # | A_42_P531701 |  | **-1,40** |
| # | # | # | # | # | Olr837_predicted | Olfactory receptor 837 (predicted) | **-1,40** |
| # | # | # | # | # | CB548191 | GLI-Kruppel family member GLI1 | **-1,40** |
| # | # | # |  | # | Hook3 | Hook homolog 3 | **-1,40** |
| # | # | # | # | # | Sept1_predicted | Septin 1 (predicted) | **-1,40** |
| # | # | # | # | # | XM_575357 | A kinase (PRKA) anchor protein (yotiao) 9 | **-1,40** |
| # | # | # |  | # | Pla2g2c | Phospholipase A2, group 2C | **-1,40** |
| # | # | # | # | # | TC520201 |  | **-1,40** |
| # | # | # |  | # | Mmaa_predicted | Methylmalonic aciduria (cobalamin deficiency) type A (predicted) | **-1,40** |
| # | # | # | # | # | LOC313060 | Similar to SPOC domain containing 1 | **-1,40** |
| # | # | # | # | # | NM_001014091 | Similar to hypothetical protein | **-1,40** |
| # | # | # | # | # | Grm5 | Glutamate receptor, metabotropic 5 | **-1,40** |
| # | # | # | # | # | LOC363167 | Similar to hypothetical protein A730098P15 | **-1,40** |
| # | # | # | # | # | TC522736 | Unknown | **-1,40** |
| # | # | # |  | # | A_43_P14091 | Unknown | **-1,40** |
| # | # |  | # | # | LOC363445 | Similar to ubiquitin specific protease 9, X-linked (fat facets-like, Drosophila) | **-1,40** |
| # | # | # |  | # | AA859319 | Transcribed locus | **-1,40** |
| # | # | # | # | # | Arfip1 | ADP-ribosylation factor interacting protein 1 | **-1,40** |


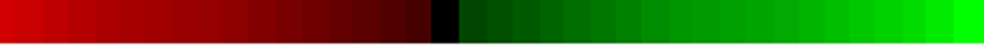


5 1.2 -1.2 -5

Hybridization was performed with samples from 3 independent experiments (E1, E2, E3) and experiment 1 with dye-swap (1DS). FC: mean fold change

**Table S4. Progestin-dependent down-regulated gene expression pattern.** The table shows individual fold changes of statistical down-regulated genes after 45 min treatment with R5020 10^-10^M related to vehicle. Down (green) regulated genes are ordered by increasing mean fold change. Data shown as indicated in Table S3.
