# Supplementary figures and images for: Correction: CDC2 Mediates Progestin Initiated Endometrial Stromal Cell Proliferation: A PR Signaling to Gene Expression Independently of Its Binding to Chromatin
Source: PLoS One. 2025 Apr 3;20(4):e0321979. doi: 10.1371/journal.pone.0321979 (PMC11967920; doi:10.1371/journal.pone.0321979)

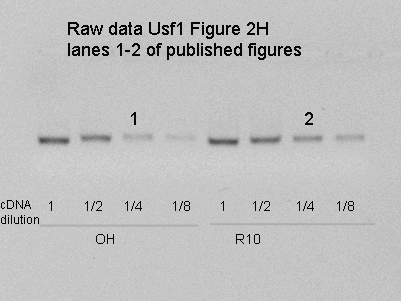

Supplement: S1 File — This includes the underlying Ccnd1 and beta Actin agarose gels from Fig 1A, the underlying Ccnd1 (RU, ICI, PD and LY) and beta Actin (RU, ICI, PD and LY) agarose gels from Fig 1C, the underlying c-Myc, Cdkn1a, beta Actin and Ccnd1 agarose gels from Fig 1D, the underlying JunD, Usf1, Crebbp, Cyr61 and Cdkn1b agarose gels from Fig 2H, and the underlying Cdc2 and Ccnb1agarose gels from Fig 4B. (ZIP) [file pone.0321979.s001.zip › 23 Figure 2H Usf1 raw data (1).tif]

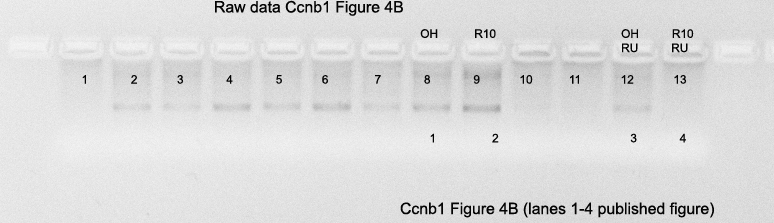

Supplement: S1 File — This includes the underlying Ccnd1 and beta Actin agarose gels from Fig 1A, the underlying Ccnd1 (RU, ICI, PD and LY) and beta Actin (RU, ICI, PD and LY) agarose gels from Fig 1C, the underlying c-Myc, Cdkn1a, beta Actin and Ccnd1 agarose gels from Fig 1D, the underlying JunD, Usf1, Crebbp, Cyr61 and Cdkn1b agarose gels from Fig 2H, and the underlying Cdc2 and Ccnb1agarose gels from Fig 4B. (ZIP) [file pone.0321979.s001.zip › 29 Figure 4B Ccnb1 raw data (1).tif]

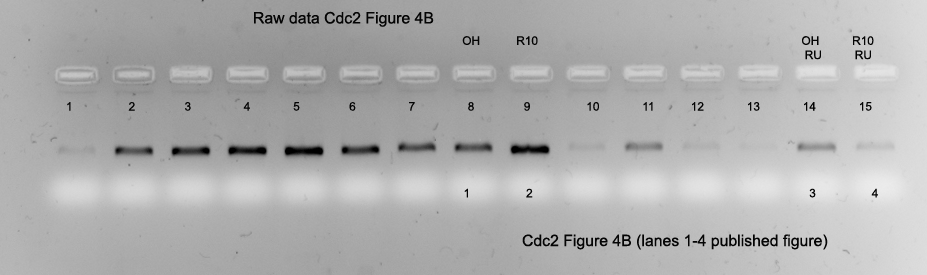

Supplement: S1 File — This includes the underlying Ccnd1 and beta Actin agarose gels from Fig 1A, the underlying Ccnd1 (RU, ICI, PD and LY) and beta Actin (RU, ICI, PD and LY) agarose gels from Fig 1C, the underlying c-Myc, Cdkn1a, beta Actin and Ccnd1 agarose gels from Fig 1D, the underlying JunD, Usf1, Crebbp, Cyr61 and Cdkn1b agarose gels from Fig 2H, and the underlying Cdc2 and Ccnb1agarose gels from Fig 4B. (ZIP) [file pone.0321979.s001.zip › 28 Figure 4B Cdc2 raw data (1).tif]

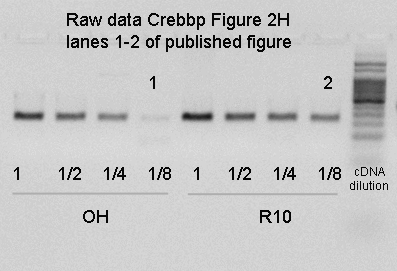

Supplement: S1 File — This includes the underlying Ccnd1 and beta Actin agarose gels from Fig 1A, the underlying Ccnd1 (RU, ICI, PD and LY) and beta Actin (RU, ICI, PD and LY) agarose gels from Fig 1C, the underlying c-Myc, Cdkn1a, beta Actin and Ccnd1 agarose gels from Fig 1D, the underlying JunD, Usf1, Crebbp, Cyr61 and Cdkn1b agarose gels from Fig 2H, and the underlying Cdc2 and Ccnb1agarose gels from Fig 4B. (ZIP) [file pone.0321979.s001.zip › 24 Figure 2H Crebbp raw data (1).tif]

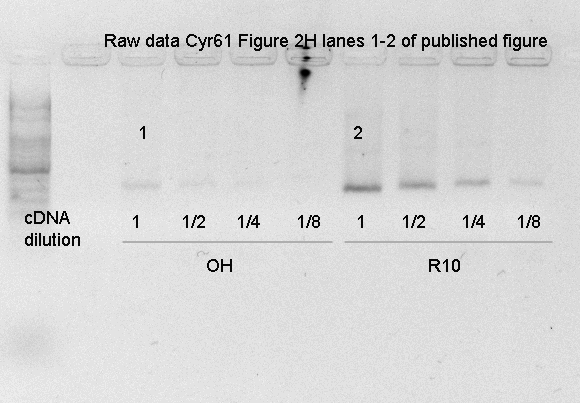

Supplement: S1 File — This includes the underlying Ccnd1 and beta Actin agarose gels from Fig 1A, the underlying Ccnd1 (RU, ICI, PD and LY) and beta Actin (RU, ICI, PD and LY) agarose gels from Fig 1C, the underlying c-Myc, Cdkn1a, beta Actin and Ccnd1 agarose gels from Fig 1D, the underlying JunD, Usf1, Crebbp, Cyr61 and Cdkn1b agarose gels from Fig 2H, and the underlying Cdc2 and Ccnb1agarose gels from Fig 4B. (ZIP) [file pone.0321979.s001.zip › 25 Figure 2H Cyr61 raw data (1).tif]

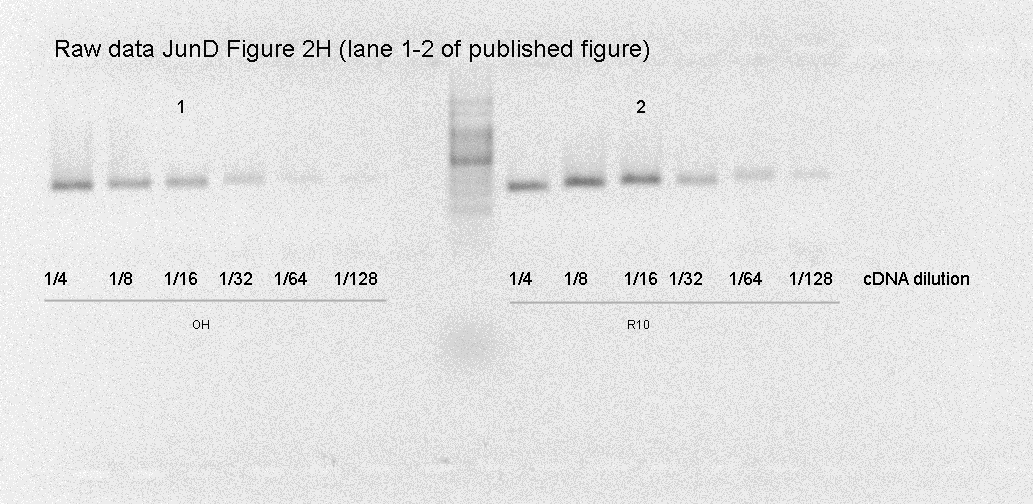

Supplement: S1 File — This includes the underlying Ccnd1 and beta Actin agarose gels from Fig 1A, the underlying Ccnd1 (RU, ICI, PD and LY) and beta Actin (RU, ICI, PD and LY) agarose gels from Fig 1C, the underlying c-Myc, Cdkn1a, beta Actin and Ccnd1 agarose gels from Fig 1D, the underlying JunD, Usf1, Crebbp, Cyr61 and Cdkn1b agarose gels from Fig 2H, and the underlying Cdc2 and Ccnb1agarose gels from Fig 4B. (ZIP) [file pone.0321979.s001.zip › 22 Figure 2H JunD raw data (1).tif]

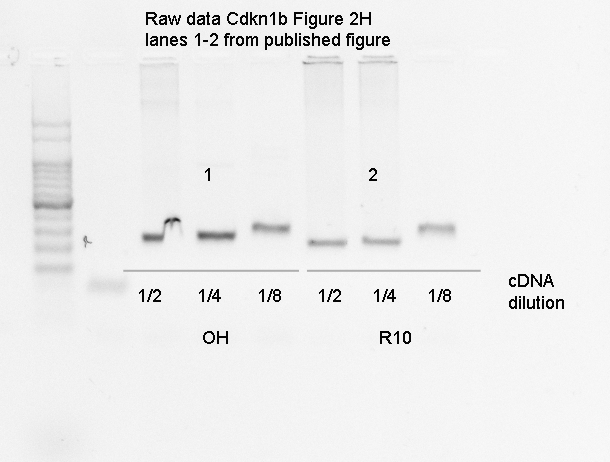

Supplement: S1 File — This includes the underlying Ccnd1 and beta Actin agarose gels from Fig 1A, the underlying Ccnd1 (RU, ICI, PD and LY) and beta Actin (RU, ICI, PD and LY) agarose gels from Fig 1C, the underlying c-Myc, Cdkn1a, beta Actin and Ccnd1 agarose gels from Fig 1D, the underlying JunD, Usf1, Crebbp, Cyr61 and Cdkn1b agarose gels from Fig 2H, and the underlying Cdc2 and Ccnb1agarose gels from Fig 4B. (ZIP) [file pone.0321979.s001.zip › 26 Figure 2H Cdkn1b raw data (1).tif]

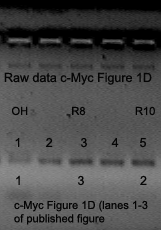

Supplement: S1 File — This includes the underlying Ccnd1 and beta Actin agarose gels from Fig 1A, the underlying Ccnd1 (RU, ICI, PD and LY) and beta Actin (RU, ICI, PD and LY) agarose gels from Fig 1C, the underlying c-Myc, Cdkn1a, beta Actin and Ccnd1 agarose gels from Fig 1D, the underlying JunD, Usf1, Crebbp, Cyr61 and Cdkn1b agarose gels from Fig 2H, and the underlying Cdc2 and Ccnb1agarose gels from Fig 4B. (ZIP) [file pone.0321979.s001.zip › 15 Figure 1D c-Myc B raw data (2).tif]

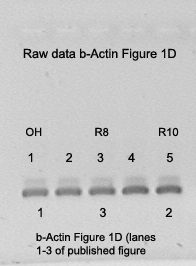

Supplement: S1 File — This includes the underlying Ccnd1 and beta Actin agarose gels from Fig 1A, the underlying Ccnd1 (RU, ICI, PD and LY) and beta Actin (RU, ICI, PD and LY) agarose gels from Fig 1C, the underlying c-Myc, Cdkn1a, beta Actin and Ccnd1 agarose gels from Fig 1D, the underlying JunD, Usf1, Crebbp, Cyr61 and Cdkn1b agarose gels from Fig 2H, and the underlying Cdc2 and Ccnb1agarose gels from Fig 4B. (ZIP) [file pone.0321979.s001.zip › 18 Figure 1D Actin raw data (1).tif]

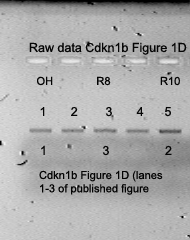

Supplement: S1 File — This includes the underlying Ccnd1 and beta Actin agarose gels from Fig 1A, the underlying Ccnd1 (RU, ICI, PD and LY) and beta Actin (RU, ICI, PD and LY) agarose gels from Fig 1C, the underlying c-Myc, Cdkn1a, beta Actin and Ccnd1 agarose gels from Fig 1D, the underlying JunD, Usf1, Crebbp, Cyr61 and Cdkn1b agarose gels from Fig 2H, and the underlying Cdc2 and Ccnb1agarose gels from Fig 4B. (ZIP) [file pone.0321979.s001.zip › 17 Figure 1D Cdkn1a raw data (2).tif]

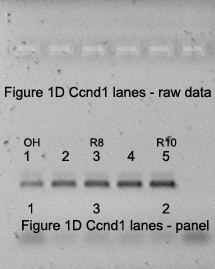

Supplement: S1 File — This includes the underlying Ccnd1 and beta Actin agarose gels from Fig 1A, the underlying Ccnd1 (RU, ICI, PD and LY) and beta Actin (RU, ICI, PD and LY) agarose gels from Fig 1C, the underlying c-Myc, Cdkn1a, beta Actin and Ccnd1 agarose gels from Fig 1D, the underlying JunD, Usf1, Crebbp, Cyr61 and Cdkn1b agarose gels from Fig 2H, and the underlying Cdc2 and Ccnb1agarose gels from Fig 4B. (ZIP) [file pone.0321979.s001.zip › 19 Figure1D Ccnd1 Raw data (1).tif]

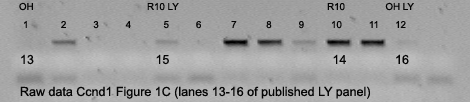

Supplement: S1 File — This includes the underlying Ccnd1 and beta Actin agarose gels from Fig 1A, the underlying Ccnd1 (RU, ICI, PD and LY) and beta Actin (RU, ICI, PD and LY) agarose gels from Fig 1C, the underlying c-Myc, Cdkn1a, beta Actin and Ccnd1 agarose gels from Fig 1D, the underlying JunD, Usf1, Crebbp, Cyr61 and Cdkn1b agarose gels from Fig 2H, and the underlying Cdc2 and Ccnb1agarose gels from Fig 4B. (ZIP) [file pone.0321979.s001.zip › 11 Figure1C Ccnd1 lanes 13to16 raw data (1).tif]

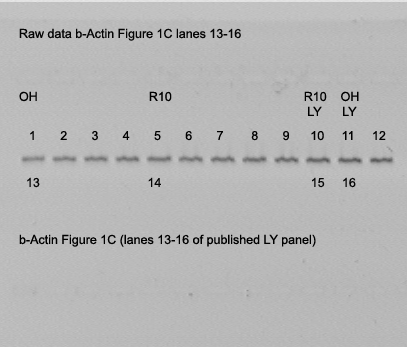

Supplement: S1 File — This includes the underlying Ccnd1 and beta Actin agarose gels from Fig 1A, the underlying Ccnd1 (RU, ICI, PD and LY) and beta Actin (RU, ICI, PD and LY) agarose gels from Fig 1C, the underlying c-Myc, Cdkn1a, beta Actin and Ccnd1 agarose gels from Fig 1D, the underlying JunD, Usf1, Crebbp, Cyr61 and Cdkn1b agarose gels from Fig 2H, and the underlying Cdc2 and Ccnb1agarose gels from Fig 4B. (ZIP) [file pone.0321979.s001.zip › 12 Figure1C Actin lanes 13to16 raw data (1).tif]

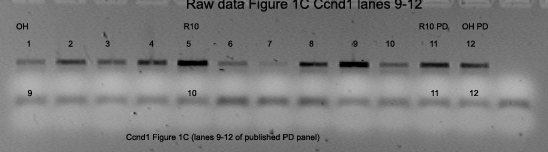

Supplement: S1 File — This includes the underlying Ccnd1 and beta Actin agarose gels from Fig 1A, the underlying Ccnd1 (RU, ICI, PD and LY) and beta Actin (RU, ICI, PD and LY) agarose gels from Fig 1C, the underlying c-Myc, Cdkn1a, beta Actin and Ccnd1 agarose gels from Fig 1D, the underlying JunD, Usf1, Crebbp, Cyr61 and Cdkn1b agarose gels from Fig 2H, and the underlying Cdc2 and Ccnb1agarose gels from Fig 4B. (ZIP) [file pone.0321979.s001.zip › 9 Figure 1C Ccnd1 lanes 9-12 raw data (1).tif]

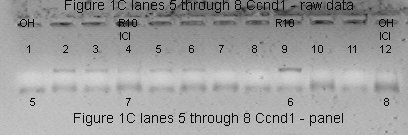

Supplement: S1 File — This includes the underlying Ccnd1 and beta Actin agarose gels from Fig 1A, the underlying Ccnd1 (RU, ICI, PD and LY) and beta Actin (RU, ICI, PD and LY) agarose gels from Fig 1C, the underlying c-Myc, Cdkn1a, beta Actin and Ccnd1 agarose gels from Fig 1D, the underlying JunD, Usf1, Crebbp, Cyr61 and Cdkn1b agarose gels from Fig 2H, and the underlying Cdc2 and Ccnb1agarose gels from Fig 4B. (ZIP) [file pone.0321979.s001.zip › 7 Figure 1C Ccnd1 lanes 5to8 raw data (2) (1).tif]

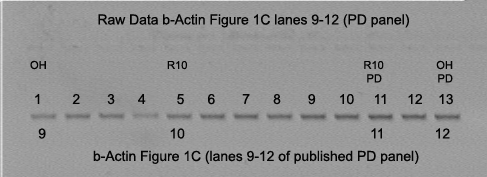

Supplement: S1 File — This includes the underlying Ccnd1 and beta Actin agarose gels from Fig 1A, the underlying Ccnd1 (RU, ICI, PD and LY) and beta Actin (RU, ICI, PD and LY) agarose gels from Fig 1C, the underlying c-Myc, Cdkn1a, beta Actin and Ccnd1 agarose gels from Fig 1D, the underlying JunD, Usf1, Crebbp, Cyr61 and Cdkn1b agarose gels from Fig 2H, and the underlying Cdc2 and Ccnb1agarose gels from Fig 4B. (ZIP) [file pone.0321979.s001.zip › 10 Figure 1C Actin lanes 9to12 raw data (1).tif]

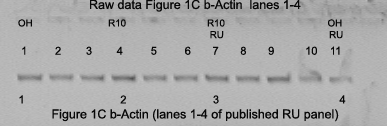

Supplement: S1 File — This includes the underlying Ccnd1 and beta Actin agarose gels from Fig 1A, the underlying Ccnd1 (RU, ICI, PD and LY) and beta Actin (RU, ICI, PD and LY) agarose gels from Fig 1C, the underlying c-Myc, Cdkn1a, beta Actin and Ccnd1 agarose gels from Fig 1D, the underlying JunD, Usf1, Crebbp, Cyr61 and Cdkn1b agarose gels from Fig 2H, and the underlying Cdc2 and Ccnb1agarose gels from Fig 4B. (ZIP) [file pone.0321979.s001.zip › 6 Figure1C Actin lanes 1to4 raw data label correction (1).tif]

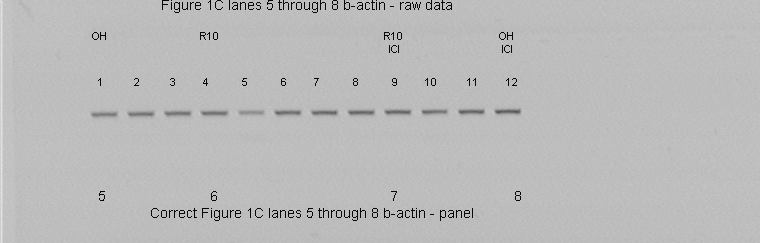

Supplement: S1 File — This includes the underlying Ccnd1 and beta Actin agarose gels from Fig 1A, the underlying Ccnd1 (RU, ICI, PD and LY) and beta Actin (RU, ICI, PD and LY) agarose gels from Fig 1C, the underlying c-Myc, Cdkn1a, beta Actin and Ccnd1 agarose gels from Fig 1D, the underlying JunD, Usf1, Crebbp, Cyr61 and Cdkn1b agarose gels from Fig 2H, and the underlying Cdc2 and Ccnb1agarose gels from Fig 4B. (ZIP) [file pone.0321979.s001.zip › 8 Figure 1C Actin lanes 5to8 raw data (1).tif]

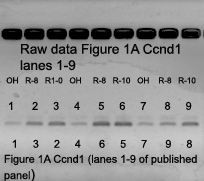

Supplement: S1 File — This includes the underlying Ccnd1 and beta Actin agarose gels from Fig 1A, the underlying Ccnd1 (RU, ICI, PD and LY) and beta Actin (RU, ICI, PD and LY) agarose gels from Fig 1C, the underlying c-Myc, Cdkn1a, beta Actin and Ccnd1 agarose gels from Fig 1D, the underlying JunD, Usf1, Crebbp, Cyr61 and Cdkn1b agarose gels from Fig 2H, and the underlying Cdc2 and Ccnb1agarose gels from Fig 4B. (ZIP) [file pone.0321979.s001.zip › 1 Figure 1A Ccnd1 raw data (1).tif]

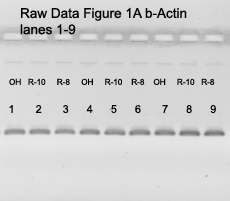

Supplement: S1 File — This includes the underlying Ccnd1 and beta Actin agarose gels from Fig 1A, the underlying Ccnd1 (RU, ICI, PD and LY) and beta Actin (RU, ICI, PD and LY) agarose gels from Fig 1C, the underlying c-Myc, Cdkn1a, beta Actin and Ccnd1 agarose gels from Fig 1D, the underlying JunD, Usf1, Crebbp, Cyr61 and Cdkn1b agarose gels from Fig 2H, and the underlying Cdc2 and Ccnb1agarose gels from Fig 4B. (ZIP) [file pone.0321979.s001.zip › 2 Figure 1A Actin Raw data (1).tif]

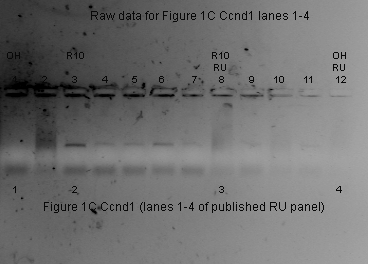

Supplement: S1 File — This includes the underlying Ccnd1 and beta Actin agarose gels from Fig 1A, the underlying Ccnd1 (RU, ICI, PD and LY) and beta Actin (RU, ICI, PD and LY) agarose gels from Fig 1C, the underlying c-Myc, Cdkn1a, beta Actin and Ccnd1 agarose gels from Fig 1D, the underlying JunD, Usf1, Crebbp, Cyr61 and Cdkn1b agarose gels from Fig 2H, and the underlying Cdc2 and Ccnb1agarose gels from Fig 4B. (ZIP) [file pone.0321979.s001.zip › 4 Figure1C Ccnd1 lanes 1to4 raw data (1) (1).tif]
